# Improved Analysis of Long-Term Monitoring Data Demonstrates Marked Regional Declines of Bat Populations in the Eastern United States

**DOI:** 10.1371/journal.pone.0065907

**Published:** 2013-06-21

**Authors:** Thomas E. Ingersoll, Brent J. Sewall, Sybill K. Amelon

**Affiliations:** 1 National Institute for Mathematical and Biological Synthesis, University of Tennessee, Knoxville, Tennessee, United States of America; 2 Department of Biology, Temple University, Philadelphia, Pennsylvania, United States of America; 3 Northern Research Station, U.S. Forest Service, University of Missouri, Columbia, Missouri, United States of America; University of Sydney, Australia

## Abstract

Bats are diverse and ecologically important, but are also subject to a suite of severe threats. Evidence for localized bat mortality from these threats is well-documented in some cases, but long-term changes in regional populations of bats remain poorly understood. Bat hibernation surveys provide an opportunity to improve understanding, but analysis is complicated by bats' cryptic nature, non-conformity of count data to assumptions of traditional statistical methods, and observation heterogeneities such as variation in survey timing. We used generalized additive mixed models (GAMMs) to account for these complicating factors and to evaluate long-term, regional population trajectories of bats. We focused on four hibernating bat species – little brown myotis (*Myotis lucifugus*), tri-colored bat (*Perimyotis subflavus*), Indiana myotis (*M. sodalis*), and northern myotis (*M. septentrionalis*) – in a four-state region of the eastern United States during 1999–2011.

Our results, from counts of nearly 1.2 million bats, suggest that cumulative declines in regional relative abundance by 2011 from peak levels were 71% (with 95% confidence interval of ±11%) in *M. lucifugus*, 34% (±38%) in *P. subflavus*, 30% (±26%) in *M. sodalis*, and 31% (±18%) in *M. septentrionalis*. The *M. lucifugus* population fluctuated until 2004 before persistently declining, and the populations of the other three species declined persistently throughout the study period. Population trajectories suggest declines likely resulted from the combined effect of multiple threats, and indicate a need for enhanced conservation efforts. They provide strong support for a change in the IUCN Red List conservation status in *M. lucifugus* from Least Concern to Endangered within the study area, and are suggestive of a need to change the conservation status of the other species. Our modeling approach provided estimates of uncertainty, accommodated non-linearities, and controlled for observation heterogeneities, and thus has wide applicability for evaluating population trajectories in other wildlife species.

## Introduction

Bats are the focus of intense conservation interest [1] due to their high levels of species diversity [2], their crucial roles in the functioning of ecological communities [3], [4], and the valuable ecosystem services they provide to people [5], [6]. Despite this conservation importance, bats are subject to a suite of severe threats [7], [8], [9], including disturbance and altered microclimates of critical hibernacula and day roosts [10], [11], [12], loss and modification of foraging areas [9], [13], [14], toxicity and changed prey composition and abundances from pesticide use and other chemical compounds [15], [16], climate change [17], [18], and in-flight collisions with vehicles, buildings, and wind turbines [19], [20], [21]. In addition, an important emerging threat to bats in eastern North America [22] with potential to spread across the continent [23], is white-nose syndrome, a disease of hibernating bats caused by a newly-discovered fungal pathogen (*Geomyces destructans*) [24]. Bats are often subject to more than one of these threats simultaneously; such co-occurring threats may result in synergistic or interacting effects, with impacts more severe than from any single threat in isolation [25], [26], [27], [28]. Combined with bats' long generation times and low reproductive rates [29], mortality from these pervasive threats has raised concern about the continued persistence of regional populations of several bat species [10], [30], [31], [32], [33] and has highlighted a critical need to improve understanding of the large-scale population dynamics of North American bat species.

Evidence of local impacts of these threats on bats – such as carcasses found in hibernacula after infection with white-nose syndrome or beneath turbines in wind farms – is well documented in some cases (e.g., [34], [35]). However, local mortality events – even when repeated over time or observed at many sites – do not necessarily indicate sustained, large-scale declines in bat abundance. This is because local mortality events may simply not be large or frequent enough to substantially affect regional populations [36], because affected populations may compensate for mortality with increases in reproduction or immigration [37], or because population declines in one locality may be offset by increases in other localities [38]. For these reasons, and because of high spatial and temporal variability in local population estimates (P. de Valpine, T.E. Ingersoll & W. Rainey, unpublished), long-term, regional estimates of abundance are essential to improving understanding of bat populations [39].

Bats' natural history and cryptic nature make them difficult to monitor, and efforts to evaluate changes in the abundance of bat species over large spatial and temporal scales have proven challenging [40], [41]. Challenges associated with censusing bats result in part from their metapopulation structure and wide range of roosting and behavioral characteristics. Mark-recapture methods, for instance, have generally proven unsuccessful for censusing bat colonies, largely because colonies (and bat populations as a whole) are not “closed”, bats are rarely recaptured, and the process of capture may lead to roost switching [41], [42]. Hibernation surveys have been more consistently used to estimate bat populations because hibernating colonies are relatively easily located and reasonably permanent; many species form large aggregated clusters during hibernation, the bats are relatively inactive making counting more feasible, and many species exhibit fidelity to particular hibernacula [43]. Population monitoring and status determination for threatened and endangered species of bats has therefore relied extensively on hibernacula surveys [44], [45], and many wildlife agencies have regularly counted bats along repeatable, well-established routes in hibernacula where bats are easy to observe and where colonies are believed to represent the population at large. Thus, the most consistently-sampled, long-term, and regional-scale data for bats in North America are from surveys of bat hibernacula completed in caves and mines as part of wildlife monitoring programs by state wildlife agencies. Data from such state-led programs for hibernacula monitoring are critical to understanding population changes in hibernating bat species.

Despite the advantages of hibernation surveys over other existing data sources for North American bats, the raw data produced by hibernation surveys are not perfectly suited to estimating changes in long-term regional populations. For instance, long-term, large-scale monitoring efforts led by multiple independent agencies may vary spatially in scope, focus, and available resources, and vary temporally with changing funding environments, turnovers in personnel, and shifting conservation priorities [46], [47]. Together such factors may result in intermittent surveys, unequal survey effort, or other observation errors which complicate the estimation of long-term, regional changes in abundance [48], [49], [50]. Bat counts from hibernacula surveys also suffer from biases associated with highly variable cluster densities of hibernating bats and detection challenges associated with varied wall and ceiling textures and contours and fissures in hibernacula [10], [51]. Estimation of abundance from bat counts is further complicated by the tendency of such counts to exhibit highly variable, irruptive patterns resulting from both actual changes in abundance and observation error (P. de Valpine, T.E. Ingersoll & W. Rainey, unpublished).

The use of wildlife counts to understand long-term changes in regional abundance also poses several additional, sometimes-unrecognized statistical challenges [52]. These challenges include the use of count data that do not conform to assumptions of traditional statistical methods, including non-Gaussian and correlated errors [53] and non-linearity [54]. Other major hindrances to monitoring population trends using hibernation surveys are the lack of knowledge of all hibernacula locations, infeasibility of monitoring all known sites in a short time frame and the complications associated with making unbiased estimates of bats (i.e., count methods that account for detection probability are warranted but have not been used extensively to date). The potential presence of unknown colonies makes it necessary to assume that trends observed in known colonies are representative of all colonies. This assumption may or may not be valid and it may introduce unknown variation in population trend assessments. Thus, although hibernacula counts have become widespread, estimates of long-term changes in regional bat abundance from these counts are rare, or where available often do not include measures of variance, confidence intervals, or account for detection probability [40]. The resulting uncertainty about regional population trends has hindered managers' ability to accurately assess bat conservation status, efficiently allocate scarce management resources to high-priority species, and develop effective management strategies [39].

A particular concern when using hibernation surveys to estimate long-term changes in bat abundance is variation in survey timing during the hibernation season [43]. Hibernation surveys are often time-consuming, and with limited available personnel and demands from various other competing conservation priorities, state agencies may stagger surveys across a hibernation season or change the dates at which surveys were conducted among years. Thus, bat counts may reflect not only year-to-year changes in abundance due to annual births, deaths, and migration, but also within-season changes in abundance or detectability. Such within-season changes may be important, especially if they represent systematic shifts in survey date over time, as survey date reflects progressive mortality over the hibernation period [24], as well as within-season changes in bat detection due to timing of fall arrival or spring emergence from hibernacula [55], winter movement within a hibernaculum between states of unequal observability (e.g., movement between ceiling surfaces and fissures, changes in packing density of clusters, or between entrance rooms and remote rooms to access preferred thermal microenvironments) [56], or winter movement between hibernacula [57]. Thus, the variable or inconsistent timing of surveys during the hibernation season could generate detection inconsistency and bias long-term estimates, even when surveying with consistent methods along standardized routes.

Statistical methods that can address these potentially complicating factors have been developed for other taxa and can be applied to bat monitoring data. First, the use of generalized models allows for the examination of data with non-Gaussian distributions of the response variable, such as count data [58], [59], [60]. Second, the incorporation of random effects in a mixed-effect model provides a means to account for correlated errors (non-independence) [53], [58]. Third, additive models permit a systematic, parsimonious examination of the variable response of relative abundance to time, and thus enable modeling of a non-linear population trajectory (enabling inference about temporal changes in trend) rather than solely assuming a linear population trend [52], [54]. Fourth, the use of smoothing terms may facilitate interpretation of irruptive data by providing regression between sample periods, while allowing deviation from linearity to be systematically modeled (P. de Valpine, T.E. Ingersoll & W. Rainey, unpublished). Fifth, hierarchical models can account for sampling heterogeneity due to differing sampling effort across space or time [61]. Finally, inconsistencies in the timing of bat hibernation surveys could be addressed by modeling within-season variation as a covariate (i.e., using models of within-season variation to estimate the bat count that would be expected if all data had been collected on the same date each year). Each of these elaborations can be accommodated with a statistical approach using generalized additive mixed models (GAMMs; [62]), a type of implicit-process hierarchical model that estimates non-linear variation in relative abundance over time [52], [61]. GAMMs provide an approach that is well-suited to modeling regional population trajectories of species across time while accounting for heterogeneous observation processes.

Our objective in this study was to improve understanding of temporal changes in regional bat abundance in a four-state region in the eastern United States. Specifically, we examined two principal research questions. First, how do inconsistencies in the timing of sampling within a season affect across-year estimates of regional bat abundance? And second, have trends in relative abundance of hibernating bats in the eastern United States changed at the regional scale in recent years? We focused on four hibernating bat species for which we could obtain data sufficient to model regional changes over time: the little brown myotis (*Myotis lucifugus*), the tri-colored bat (*Perimyotis subflavus*), the Indiana myotis (*M. sodalis*), and the northern myotis (*M. septentrionalis*). We focused on changes in the abundance of these species in the region comprising New York, Pennsylvania, West Virginia, and Tennessee during a 13-year period.

## Methods

### Data collection

We obtained data on the four focal bat species from state agencies in New York, Pennsylvania, West Virginia, and Tennessee. Data were from hibernation surveys completed during 1999–2011 by trained biologists as part of long-term wildlife-monitoring programs. Surveys were performed during the hibernation period from December-March in caves and mines known to serve as bat hibernacula for one or more bat species. Out of concern for negative effects of disturbing hibernating bats [12], and due to limited personnel and resources for monitoring, hibernation surveys were typically conducted once every two to three years [42]. In simple hibernacula, most accessible areas of the hibernaculum were surveyed during a single visit. In complex hibernacula, surveys were restricted to specified survey routes. Very large, complex hibernacula were divided into multiple survey routes. During surveys, bats were visually counted where they hibernated on walls and ceilings [42], [43]. In some cases, large clusters were photographed during surveys and later counted from photographs to minimize disturbance and improve accuracy of bat counts [43].

### Datasets

From the full datasets provided by state agencies, which comprised data from more than 636 surveys along 163 survey routes, we selected those surveys that provided the most reliable, consistently-collected count data on hibernating bats. On the basis of survey notes, we excluded all surveys that were incomplete or inconsistent, that were focused on recording incidence of white-nose syndrome in hibernacula rather than counting hibernating bats, or that deviated from established survey routes. We also excluded routes for which only a single survey remained in the dataset. The resulting dataset included a mean of 3.95 (range: 2–10) surveys per route. Because of the potential influence of survey timing on bat counts (see Introduction), we examined the extent of within-season and across-year variation in the timing of hibernation surveys with boxplots.

We examined the dataset separately for each bat species. To reduce zero-inflation due to inclusion of unsuitable habitat in the dataset, we excluded routes in which the focal bat species was never observed. This rendered a sample of 577 surveys along 145 routes counting 982974 individual *M. lucifugus*, 576 surveys along 145 routes counting 68148 individual *P. subflavus*, 284 surveys along 62 routes counting 136386 individual *M. sodalis*, and 460 surveys along 109 routes counting 5206 individual *M. septentrionalis*. Data are provided in Appendix S1.

### Global model

To evaluate temporal changes in regional abundance, we identified a global mixed-effects model *a priori* [63]. Route terms were assigned to random effects, to represent our sample within this population [53], [58]. Since detection probability was not explicitly estimated and metapopulation boundaries were unknown, data from hibernacula surveys represent an unknown fraction of the population rather than a complete census [43]. We therefore limited inference to relative, rather than absolute abundance [61]. To represent within-season and year-to-year variation, this model included fixed-effects terms for day-of-winter (*Day*), which indicated date of the survey in number of days since December 1, and year (*Year*). To relax assumptions of independence to accommodate hibernacula with more than one survey route, we controlled for between-site variation by nesting survey route within hibernaculum with a random grouping term [58] and modeled unmeasured differences between hibernaculum with a random-intercept term [58], [64]. Terms for the global model are represented by the following equation:
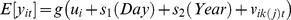
(1)

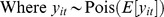


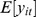
 is the expected count for species *i* at time *t*, *g* is the inverse of the selected link function (in our case the inverse of the natural logarithm *ln*), 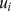 is the mean count for a species, 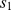 and 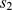 are smoothing functions for *Day* and *Year*, and 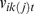 is a random effect for species *i*, at survey route *k* nested within location (hibernaculum) *j* and time *t*. The model assumed Poisson-distributed counts.

### Model selection

We then modified the global model to create a final model for each bat species, using a step-wise reduction in fixed and random effects. We evaluated the utility of including smoothing functions (cubic regression splines) to examine temporal variation in the fixed effects in the final model for each species by comparing smoothed and unsmoothed versions of the explanatory variables *Day* and *Year*. Each candidate model was generalized using a log-link and Poisson distribution. The assumption of Poisson-distributed counts was validated through graphic comparison of results for each species using Poisson models and overdispersed models using the quasi-Poisson structure. Because little distinction was evident between these models, we proceeded with the assumption of Poisson-distributed counts, which facilitated model selection. To improve model clarity and reduce the potential for over-fitting, we used smoothing functions with a maximum basis dimension that was large enough that extrema were apparent, but small enough that curvature was simple [53]. These were maximum basis dimensions larger than half the number of time steps by year, but smaller than the total number of time steps by year. We then selected the final model as the best candidate model for each species given the data with Akaike's Information Criterion (AIC; [63], [65]). This model selection process was completed with the statistical computing language *R* version 2.14.0 [66].

### Predictive GAMMs

We produced separate GAMMs from the final model for each species [54], [67] in *R* [66]. Sample *R* codes are provided in Appendix S2. Final model terms were fit using penalized quasi-likelihood methods in the *R* software package *mgcv* [68]. Library *mgcv* computes effective degrees of freedom for smoothed terms from the trace of the GAMM influence matrix, for computing AIC values [62]. We examined the within-season response of relative abundance to smoothed *Day* graphically. To examine variation across years, we extracted parameters from the GAMMs to populate predictive models, then fixed the value for *Day* at its median for each species, calculating trajectories as if they had all been sampled on the same day-of-winter. We compared trajectories with and without correction for variation in survey date. We calculated expected values and approximate confidence intervals using the *R* function *predict* [69]. Confidence intervals were estimated at plus and minus twice the standard error [62]. Because the interpretation of confidence intervals at multiple time periods within a repeated measures context is subject to debate ([52]; P. de Valpine, T.E. Ingersoll & W. Rainey, unpublished), we used the *R* function *anova* [66] to calculate p-values for the smoothed *Day* and smoothed *Year* terms, testing the null hypothesis of unchanging relative abundance over time.

Because our GAMMs estimate relative, rather than absolute abundance [61], we sought to avoid the perception that estimates of relative abundance were informative of absolute abundance. We therefore normalized estimates for 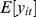 after calculating trajectories [52], providing a common scale of relative abundance for all species. To avoid selection of an arbitrary baseline year from which to normalize counts and measure population changes over time, we calculated the relative abundance of a species by dividing predicted values by the maximum expected value for that species. Thus, our normalization procedure set the maximum abundance estimate for a species during the study period equal to 1.0. Because the GAMMs produced a complex series of additive terms, predicted relative abundances and confidence intervals were rendered graphically for ease of interpretation.

To evaluate the influence of bias from within-season survey date on estimates of long-term population trajectories, we compared corrected trajectories (which accounted for variable survey date) and naïve uncorrected trajectories (where survey date was not included in the model). Corrected trajectories were from the final models for each species, and models for uncorrected trajectories excluded the fixed effect for *Day*. In one species (*P. subflavus*), *Day* was not selected in the final model (see Results), so comparison was between the final model and the best alternate model that included *Day*.

## Results

### Survey timing

**Figure 1:**
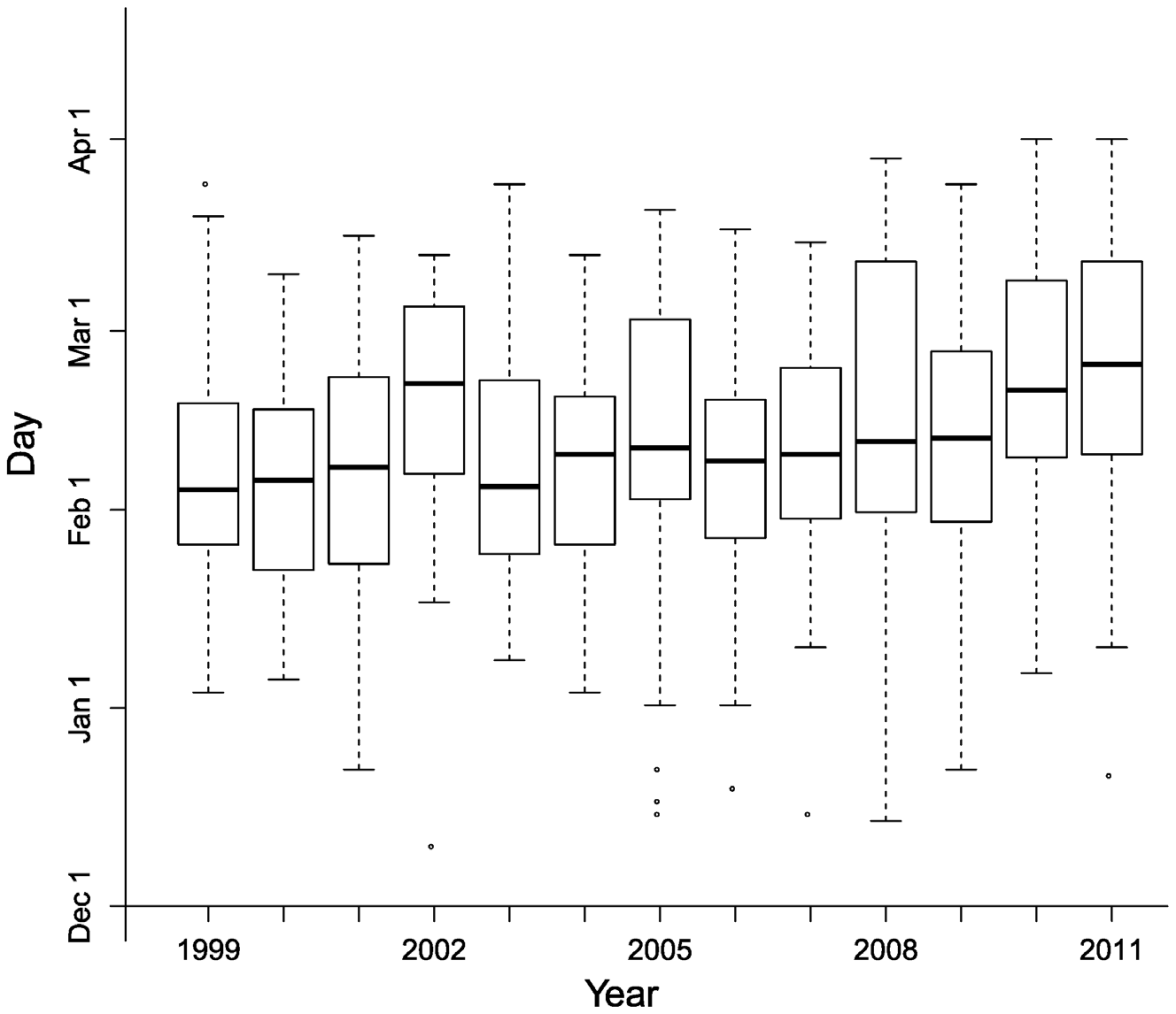
Timing of hibernation surveys across years. Box plots showing date of hibernacula surveys during 1999–2011.

The timing of hibernacula surveys varied substantially within the hibernation season in every year, and exhibited a systematic shift towards later dates (Fig. 1). Half of all surveys in each year were conducted during a 3–6 week period from late January to early March, but some surveys were conducted as early as mid-December and as late as the end of March. Within-season variability in survey date was highest in 2008. The median survey date was early February in most years, with a trend toward later date with time starting in 2006 (Fig. 1). In three years, median survey date was particularly late; ∼1 week later than other years in 2010, and ∼2 weeks later in both 2002 and 2011.

### Model selection

AIC model selection (Table 1) resulted in the selection of the fixed effects terms *Day* and *Year* for *M. lucifugus*, *M. septentrionalis* and *M. sodalis*. Smoothed *Day* was selected for these three species, smoothed *Year* was selected for *M. lucifugus* and linear *Year* was selected for *M. septentrionalis* and *M. sodalis*. For *P. subflavus*, linear *Year* alone was selected. Random intercept terms for *Route* were selected for all species, and *Route* within *Location* was selected for *M. lucifugus*.

**Table 1:** Model selection.

Species	Model	DF	AIC	Δ*_i_*	*w_i_*
A.) *M. lucifugus*					
	s(Year)+s(Day)+r(Route in Location)	8	2144.071	0	0.9998691
	Year+s(Day)+r(Route in Location)	7	2163.075	19.004	7.47E-05
	s(Year)+s(Day)+r(Route)	7	2163.644	19.573	5.62E-05
	Year+s(Day)+r(Route)	6	2181.439	37.368	7.68E-09
	s(Year)+Day+r(Route in Location)	7	2190.005	45.934	1.06E-10
	Year+Day+r(Route in Location)	6	2191.708	47.637	4.53E-11
	Year+Day+r(Route)	5	2208.487	64.416	1.03E-14
	s(Year)+s(Day)+r(Location)	7	2345.763	201.692	1.60E-44
	Year+s(Day)+r(Location)	6	2350.323	206.252	1.63E-45
	Year+Day+r(Location)	5	2359.561	215.49	1.61E-47
	s(Year)+Day+r(Location)	6	2365.796	221.725	7.13E-49
	s(Year)+Day	5	3027.745	883.674	1.30E-192
	s(Year)+Day+r(Route)	6	11396.335	9252.264	0
	Year+s(Day)	5	11639.636	9495.565	0
	Year+Day	3	3220720.178	3218576	0
	s(Year)+s(Day)	6	NA	NA	NA
B.) *P. subflavus*					
	Year+r(Route)	4	1697.105	0	0.402352
	Year+Day+r(Route)	5	1698.34	1.235	0.216985
	s(Year)+Day+r(Route in Location)	7	1699.257	2.152	0.137184
	Year+Day+r(Route in Location)	6	1700.094	2.989	0.090272
	Year+s(Day)+r(Route)	6	1701.097	3.992	0.054671
	s(Year)+s(Day)+r(Route)	7	1701.484	4.379	0.045052
	Year+s(Day)+r(Route in Location)	7	1702.857	5.752	0.022676
	s(Year)+s(Day)+r(Route in Location)	8	1703.106	6.001	0.020022
	1+r(Route)	3	1705.114	8.009	0.007336
	Day+r(Route)	4	1707.343	10.238	0.002407
	s(Year)+Day+r(Location)	6	1709.976	12.871	0.000645
	Year+Day+r(Location)	5	1712.217	15.112	0.00021
	s(Year)+s(Day)+r(Location)	7	1713.513	16.408	0.00011
	Year+s(Day)+r(Location)	6	1714.217	17.112	7.74E-05
	s(Year)+s(Day)	6	2462.475	765.37	2.55E-167
	s(Year)+Day	5	2524.623	827.518	8.15E-181
	s(Year)+Day+r(Route)	6	7283.807	5586.702	0
	Year+s(Day)	5	8027.605	6330.5	0
	Year+Day	3	155617.2	153920.1	0
C.) *M. sodalis*					
	Year+s(Day)+r(Route)	6	1196.861	0	0.705854
	Year+s(Day)+r(Route in Location)	7	1198.612	1.751	0.294097
	Year+Day+r(Route)	5	1217.254	20.393	2.63E-05
	Year+Day+r(Route in Location)	6	1219.254	22.393	9.69E-06
	s(Year)+s(Day)+r(Route in Location)	8	1219.361	22.5	9.18E-06
	s(Year)+Day+r(Route in Location)	7	1221.254	24.393	3.56E-06
	Year+Day+r(Location)	5	1396.828	199.967	2.67E-44
	s(Year)+Day+r(Location)	6	1398.828	201.967	9.82E-45
	s(Year)+Day	5	1653.314	456.453	5.39E-100
	s(Year)+s(Day)	6	1655.314	458.453	1.98E-100
	Year+s(Day)+r(Location)	6	1655.314	458.453	1.98E-100
	s(Year)+s(Day)+r(Location)	7	1657.314	460.453	7.29E-101
	s(Day)+r(Route)	5	4811.008	3614.147	0
	s(Year)+Day+r(Route)	6	4815.838	3618.977	0
	s(Day)+r(Location)	5	5053.719	3856.858	0
	s(Day)	4	5073.99	3877.129	0
	Year+s(Day)	5	5074.828	3877.967	0
	Year+Day	3	520913.1	519716.2	0
	s(Year)+s(Day)+r(Route)	6	NA	NA	NA
	s(Day)+r(Route in Location)	6	NA	NA	NA
D.) *M. septentrionalis*					
	Year+s(Day)+r(Route)	6	1884.042	0	0.963444
	Year+Day+r(Route)	5	1890.656	6.614	0.035287
	s(Year)+s(Day)+r(Route)	7	1897.422	13.38	0.001198
	s(Year)+Day+r(Route)	6	1903.063	19.021	7.14E-05
	s(Year)+s(Day)	6	2800.954	916.912	7.57E-200
	s(Year)+Day	5	2844.404	960.362	2.78E-209
	Year+s(Day)	5	5075.137	3191.095	0
	Year+Day	3	24792.18	22908.14	0

Shown are information criteria for fit of models including the fixed and random effects of (A) *M. lucifugus*, (B) *P. subflavus*, (C) *M. sodalis*, and (D) *M. septentrionalis*. Fixed effects are *Day*, smoothed *Day*, *Year*, and smoothed *Year*, and the random effects are *Route* and *Route* nested in *Location*. Best models were selected on the basis of Akaike's Information Criterion (AIC). DF are the degrees of freedom, Δ*_i_* is the difference in AIC between the top-ranked and listed model, and *w_i_* is the Akaike weight, the weight of evidence for each model in the set given the data (where 1.00 represents the highest likelihood of the model relative to other models). The number of models examined varied for each species because some random effects were not applicable for some species, due to the particular survey routes used.

### Predictive GAMMs

**Figure 2:**
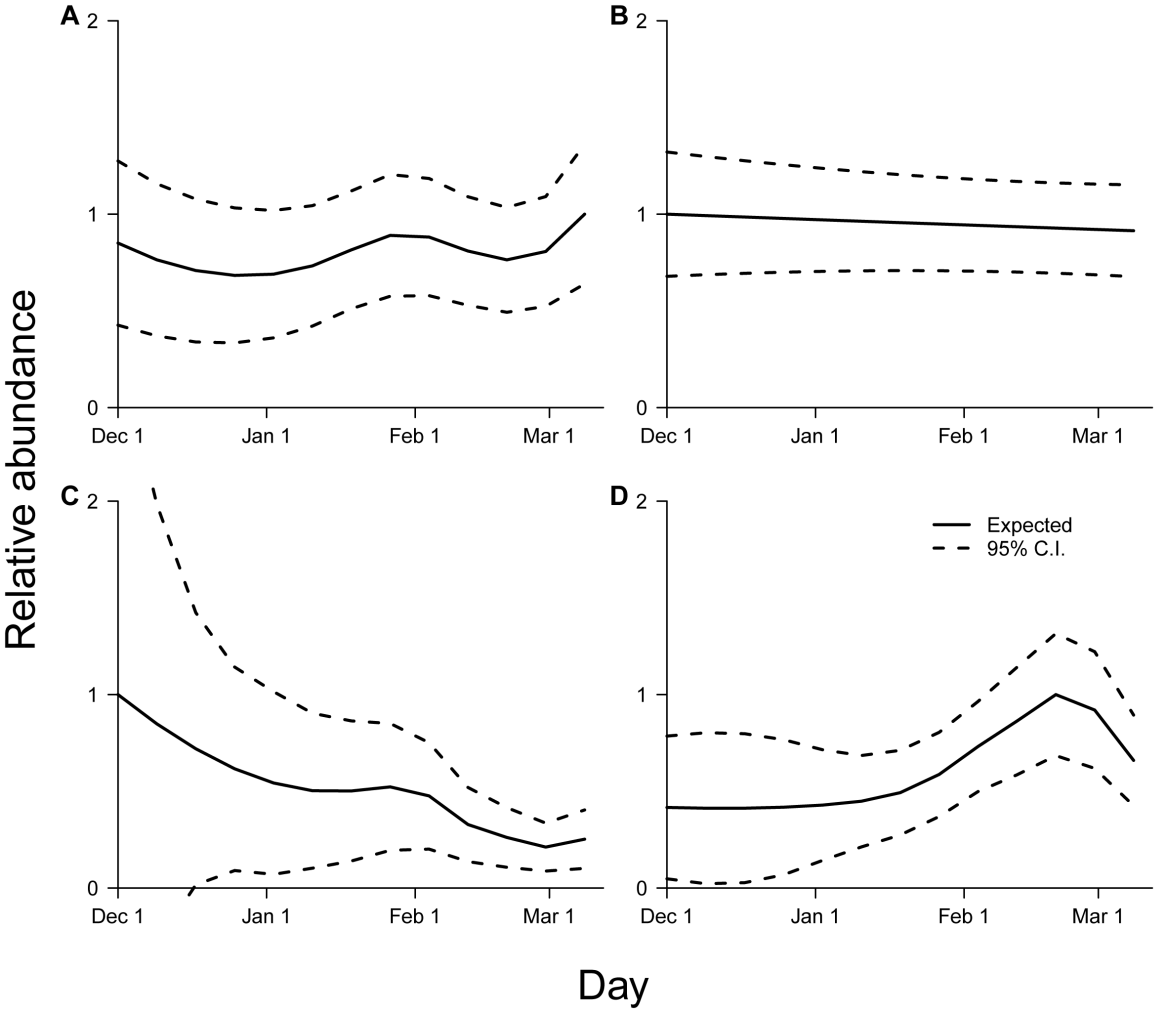
Within-season temporal variation in bat counts. Relative abundance and approximate 95% confidence intervals during December-March for (A) *M. lucifugus*, (B) *P. subflavus*, (C) *M. sodalis*, and (D) *M. septentrionalis*. Relative abundance was set equal to 1.0 at the maximum expected value.

**Figure 3:**
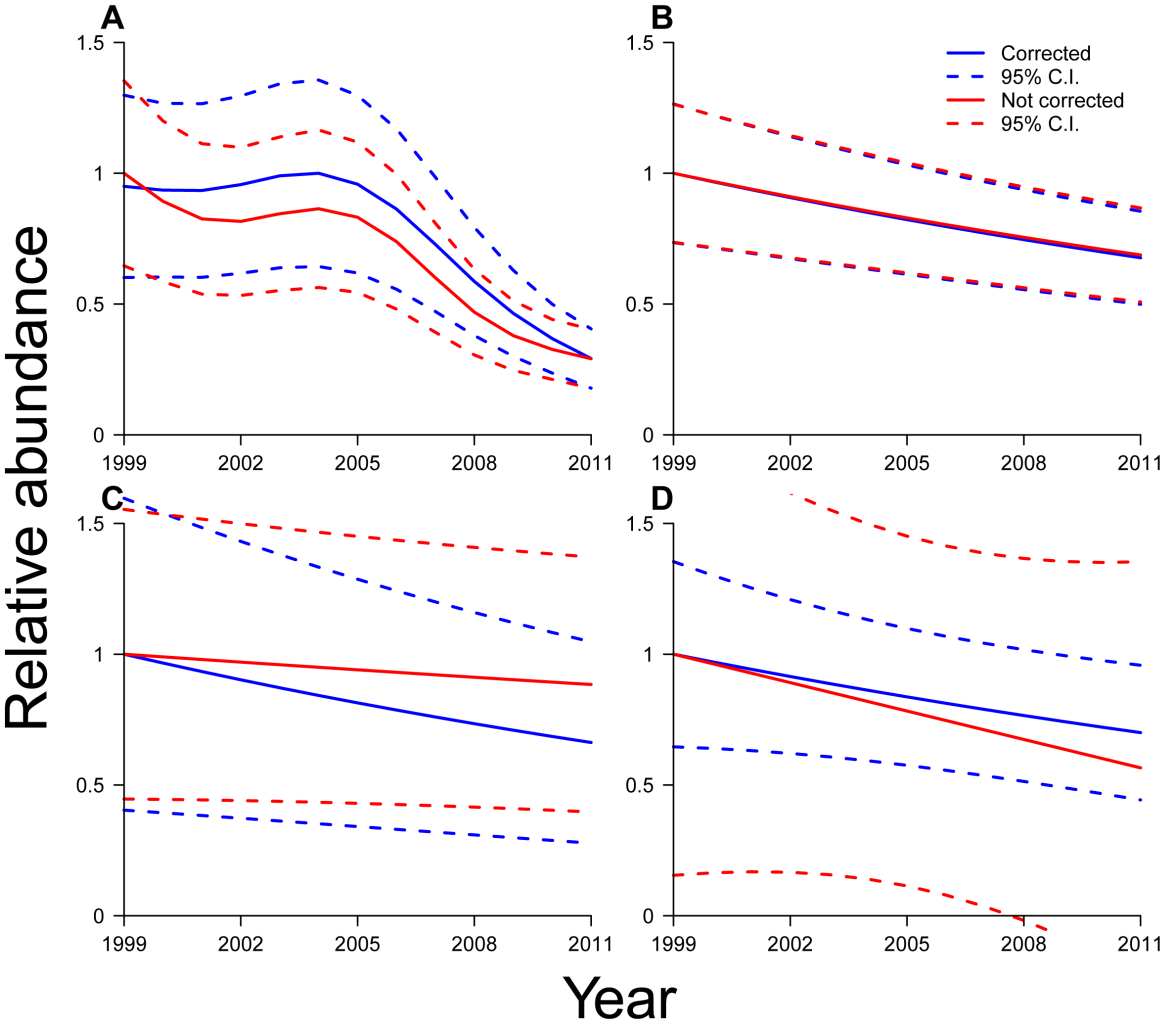
Long-term population trajectories. Expected relative abundance and approximate 95% confidence intervals during 1999–2011 for (A) *M. lucifugus*, (B) *P. subflavus*, (C) *M. sodalis*, and (D) *M. septentrionalis*. Relative abundance was set equal to 1.0 at the maximum expected value. Two trajectories are shown for each bat species: the trajectory with abundance estimates corrected for survey date of bat counts (in blue), and the uncorrected trajectory (red).

Relative abundance varied non-linearly with survey date within the hibernation season, in all species except *P. subflavus* (smoothed *Day* terms, *M. lucifugus*, F_6.48_ = 10.03, p<0.001; *M. sodalis*, F_5.90_ = 21.88, p<0.001; *M. septentrionalis*, F_4.34_ = 2.93, p = 0.033; Fig. 2; Appendix S3), suggesting that, if not accounted for, systematic heterogeneity in survey timing would bias relative abundance estimates in three of the four species. When comparing long-term trajectories corrected for survey timing (blue traces in Fig. 3) versus naïve uncorrected trajectories (red traces in Fig. 3), we found that uncorrected models underestimated relative abundance for *M. lucifugus* in most years (Fig. 3A). Declines in *M. sodalis* were underestimated in uncorrected models (Fig. 3C). Declines in *M. septentrionalis* were overestimated in uncorrected models (Fig. 3D). As expected, due to the insignificant effect of survey day for this species, trajectories from corrected versus uncorrected models for *P. subflavus* were nearly indistinguishable (Fig. 3B).

Corrected estimates of regional relative abundance demonstrated marked declines across the study period for each species (smoothed *Year* terms, *M. lucifugus*, F_4.912_ = 42.69, p<0.001; *P. subflavus*, F_1_ = 30.02, p<0.001; *M. sodalis*, F_1_ = 18.68, p<0.001; *M. septentrionalis*, F_1_ = 3.90, p = 0.049; blue traces in Fig. 3; Appendix S3, S4). Some growth in the regional population of *M. lucifugus* was evident prior to 2004 (Fig. 3A; Appendix S4). Dynamics of the final models were remarkably similar among the other three species, exhibiting slow, steady declines in all cases (Figs. 3B, 3C, 3D; Appendix S4). Cumulative declines in regional relative abundance by 2011 from peak levels were 71% (with 95% confidence interval of ±11%) in *M. lucifugus*, 34% (±38%) in *P. subflavus*, 30% (±26%) in *M. sodalis*, and 31% (±18%) in *M. septentrionalis* (Appendix S4).

## Discussion

### Population trajectories

Our results clearly show that within our four-state study area of the eastern United States, the regional populations of *M. sodalis* and *M. septentrionalis* were in decline (Fig. 3C, 3D; Appendices S3, S4) and the regional population of *M. lucifugus* was in sharp decline (Fig. 3A; Appendices S3, S4). Our data are strongly suggestive that the regional population of *P. subflavus* was also in decline (Fig. 3B; *Year* term in Appendix S3), though our estimates of decline for this species did not exceed 95% confidence intervals (Fig. 3B; Appendix S4). It is unclear why *M. lucifugus* would be declining faster than the other species, but each of these species varies in the location and environmental conditions of preferred foraging and hibernation sites [70], and these factors may be important in determining susceptibility to disturbance [12] and population-level responses to climate change [18] and disease [71]. As the most common hibernating species in this region, and one that clusters in large aggregations [70], *M. lucifugus* may also be particularly susceptible to disturbance [12] and bat-to-bat transmission of diseases such as white-nose syndrome [71], [72]. It also is the recipient of less conservation attention than the other species that aggregates in large clusters in the region, *M. sodalis*, which is federally-protected [45].

Population dynamics varied by species. We estimated that the relative abundance of *M. lucifugus* fluctuated slightly and gradually during the beginning of the study period (although still well within confidence intervals during this period), reached a peak in the study area during 2004, then declined severely and persistently thereafter (Fig. 3A; Appendix S4). Trajectories for all other species suggested a persistent declining trend throughout the study period (Fig. 3B, 3C, 3D; Appendix S3, S4). These different model dynamics may have occurred because the greater amount of data on *M. lucifugus* enabled us to determine finer-scale temporal variation in regional population trajectories. Alternatively, these dynamics may represent ecological differences among the species in response to environmental conditions or to ongoing threats. The pre-2004 increase in *M. lucifugus* could suggest a period of favorable environmental conditions against a background of overall decline, bat recovery after past declines, colonization of new winter habitat (perhaps associated with climate change), or changing levels of threat over time.

Our estimates of decline were less severe than previous estimates [22], [32]. This difference is likely because, unlike previous studies, our study analyzed data from hibernacula from across the region, including not only hibernacula infected with white-nose syndrome, but also hibernacula where white-nose syndrome had not been detected. Additionally, our estimates utilized the first multi-species, regional estimates of long-term trajectories in abundance of bat populations in North America to account for several factors believed to bias estimates of abundance from wildlife count data: non-linearity with time (Figs. 2, 3), non-Gaussian error distributions, variation among sites, and correlated errors [48], [49], [50]. In addition, although we were not able to explicitly estimate heterogeneous detection probability with these data, we did correct for an important source of detection heterogeneity [48], namely variation in sampling date (Figs. 1, 2, 3). Nonetheless, our results indicate considerable cause for concern for the regional populations of all four hibernating bat species studied. Although substantial uncertainty remains in estimates for some species, mean estimates of regional declines from their peak abundances were equal to or greater than 30% by 2011 in all species studied (Fig. 3; Appendix S4). The regional population of *M. lucifugus* is of particular concern, as mean estimates of decline from its peak abundance reached 71% by 2011 (Fig. 3A; Appendix S4). If trajectories continue along their current paths (Fig. 3), substantial further declines can be expected for each species in the near future [22]. Because of the critical ecological roles of bats in directly limiting populations of nocturnal arthropods through predation [3], [73], [74], indirectly limiting herbivory by predated species of arthropods in forests [73], [75] and agricultural systems [5], [76], and providing the nutrient subsidies [77] that are essential to supporting diverse and specialized cave fauna [78], the regional declines in the abundances of hibernating bat species that we observed could have important cascading effects in ecological communities.

### Within-season variation in abundance

Unlike previous studies, our methods corrected long-term population trajectories for within-season variation. Such correction is needed to reduce bias, especially given the high variability in dates of hibernacula surveys, and recent systematic shifts to later date-of-winter for hibernacula surveys near the end of the survey period (Fig. 1). In *P. subflavus*, survey date had almost no effect on within-season estimates of relative abundance (Table 1B; Fig. 2B), and correction did not influence long-term estimates of relative abundance (Fig. 3B). However, survey date had an important effect on within-season estimates of relative abundance in the other three species (Tables 1A, 1C, 1D; Figs. 2A, 2C, 2D). As a result, our correction for survey date substantially changed estimates of rates of decline and cumulative amounts of decline in *M. sodalis* (Fig. 3C) and *M. septentrionalis* (Fig. 3D), and changed estimates of patterns and timing of population changes (but not cumulative amounts of decline over the study period) in *M. lucifugus* (Fig. 3A).

The influence of survey date on within-season and across-year estimates of relative abundance in these bat species may arise as a result of changes in the size of colonies within hibernacula over the course of the hibernation season. Hibernation imposes severe physiological challenges on bats, including progressive energy and evaporative water loss over the hibernation period [79], [80], and these challenges are exacerbated by disturbance in hibernacula [12] and white-nose syndrome [81]. As a result, progressive mortality is expected, and should lead to a gradual reduction of the colony (and declines in regional relative abundance) over the hibernation period [24]. This could explain the within-season declines we observed throughout the hibernation period in *M. sodalis* (Fig. 2C), and the within-season declines late in the hibernation period in *M. septentrionalis* (Fig. 2D). In addition, bat species vary in the dates and duration of hibernation [55], frequency of between-hibernacula movements [57], and propensity to emerge from hibernation during intervals of warm weather during winter [57]. Variation among bat species in hibernation timing would cause a portion of the population to be present outside of the hibernacula, and thus be undetectable during hibernation surveys. Such variation could result in the non-linearities in within-season patterns of relative abundance that we observed in three of the four species in this study (Tables 1A, 1C, 1D; Figs. 2A, 2C, 2D).

The bat species' differing roosting habits during hibernation may also affect detectability, which could also help explain the influence of within-season variation in survey date on bat counts. In particular, the small size, distinctive forearms, and style of roosting away from large aggregations make *P. subflavus* easy to identify, and its habit of roosting as singles or in very small clusters hanging on walls or ceilings make them relatively easy to count throughout the hibernation season [82]. In contrast, the roosting behavior of the other three species may vary throughout the hibernation season in ways that affect detectability and thus bat counts. *M. septentrionalis* also roosts as singles or small groups, but tends to seek out crevices in walls or ceilings. Because differences in temperature or environmental factors may affect how deep they move into crevices [83], this species may vary in detectability across the hibernation season, particularly in hibernacula with high ceilings or deep crevices. *M. lucifugus* and *M. sodalis*, in contrast, often hibernate in large aggregations. Although they typically form large clusters (10's to 1000's) that are easy to locate, these clusters may vary considerably in density of packing throughout the hibernation season [82], [84], [85], perhaps also due to temperature or other environmental factors [83]. These species are also typically found on high ceilings, which makes estimating such within-season changes in cluster density difficult unless high-resolution photography is used [51].

### Threats contributing to bat population declines

It is not possible to definitively attribute population declines to specific causes solely from count data, but the timing of changes in population trajectories may be suggestive of key threats associated with these changes [52]. White-nose syndrome has often been assumed to be the key cause of regional population declines in hibernating bat species in the region we studied, due to repeated observations of mass mortality events associated with the disease [22], [32], [34], [86]. Our population trajectories, indicating persistent declines in each species in recent years (Fig. 3; Appendix S3, S4) are consistent with the interpretation that white-nose syndrome is a severe and pervasive threat that has led to extensive declines in regional bat abundance.

Our results (Fig. 3; Appendix S4), however, indicate changes in regional bat abundances throughout the study period, including declines in all species that began prior to the wide proliferation of white-nose syndrome in the region beginning in 2008–2010, and prior even to the first detection of white-nose syndrome in 2006 [22], [34]. In *M. lucifugus*, we observed declines that began in 2004, two years prior to the first recorded instance of the disease in the region [34]. AIC-based inference in which linear models were selected suggests that declines in all other species persisted across the study period, and so preceded discovery of WNS by at least seven years. Furthermore, insufficient evidence existed to support a conclusion that rates of decline increased in the other species following 2006 (Tables 1B, 1C, 1D). Mass mortality events due to white-nose syndrome are associated with large numbers of bat carcasses in hibernacula that should have been evident during hibernacula surveys [22], so it is unlikely that the epizootic went undetected for several years prior to 2006. Rather, additional persistent threats likely contributed substantially to recent declines.

Which other threats could have caused recent bat population declines remains unclear. Bat mortalities from in-flight collisions with turbines have increased with expanding wind energy development [20], [21] since 2000 [87], but these have been primarily associated with fatalities of migratory, foliage- and tree-roosting bats rather than hibernating bats [20]. Recent climate changes could also have played a role [17], [18], but climate changes have been gradual in the region for at least thirty years [18], including during periods in which bat populations were increasing (Fig. 3A). Other long-standing threats, such as changes to critical roosting [10], [11], [12] or foraging habitat [9], [13], [14], in-flight collisions with vehicles or buildings [19], or effects of pesticides and other chemical compounds [15], [16], [26] may also have influenced regional bat abundances, though it is unclear whether any single threat could have produced the persistent declines we observed (Fig. 3). The temporally-variable trajectory of *M. lucifugus* in particular may reflect synergistic or interacting effects of multiple threats [52], perhaps in conjunction with bat population responses to variable environmental conditions [17]. Further study is needed to clarify the relative importance of each threat for bat populations.

### Implications of regional declines in bat abundances for bat conservation status

*M. lucifugus*, *P. subflavus*, and *M. septentrionalis* are not currently federally-protected in the United States, whereas *M. sodalis* is listed as Endangered [88], [89], the most protected designation under the federal Endangered Species Act. Several proposals are currently being evaluated for listing the three currently-unprotected species at the state or federal level within the United States (e.g., [33], [90], [91]). We do not evaluate the merits of these proposals here. However, our results indicating multi-year, continuing declines suggest a need for enhanced conservation efforts for all four bat species, and for hibernating bat species more generally.

The IUCN Red List thresholds for assigning Vulnerable or Endangered conservation status are, respectively, 30% or 50% declines in population size, where causes of decline may not have ceased or may not be understood [92]. According to the IUCN Red List, *M. sodalis* is currently Endangered with a decreasing population trend [30], and *M. lucifugus*, *P. subflavus*, and *M. septentrionalis* all currently have an IUCN conservation status of Least Concern with a stable population trend [30]. Our estimates (Fig. 3; Appendix S3, S4), however, provide new evidence in our study area of regional-scale declines in these species, and especially in *M. lucifugus*. Specifically, in our study area, *M. lucifugus* exceeded the 30% (Vulnerable) threshold of population decline by 2008 and the 50% (Endangered) threshold by 2009. In both cases, the upper confidence limit on *M. lucifugus* abundance estimates exceeded these thresholds in the following year, indicating substantial certainty in these results. The 30% threshold of population decline was exceeded in our study area by 2010 in *P. subflavus*, and by 2011 in *M. sodalis* and *M. septentrionalis*, though upper confidence limits have not yet exceeded the 30% threshold in these three species. Note, however, that the declines in *M. sodalis* are on top of persistent historical declines that occurred in the species up through 2003 [30], [31], [89].

The implications of our regional-scale results for the conservation status of each species as a whole depend on whether the declines we observed in our four-state study area are pervasive across each species' geographic range. The species *M. sodalis* [93] and *M. septentrionalis* [94] and the subspecies *M. lucifugus lucifugus* [95] and *P. subflavus subflavus* [96] occur principally in eastern and northern North America, where many of the threats we identified above are widespread and increasing [7], [9]. This includes white-nose syndrome, which has already spread from a single known hibernaculum to 22 eastern, southeastern, and Midwestern states and five eastern Canadian provinces in only seven years [97], and has the potential to rapidly spread across the remaining entire geographic ranges of each taxon studied here [93], [94], [95], [96]. Thus our results likely have strong implications for conservation status at the species or subspecies level.

### Sampling approach

The hibernacula surveys we used in this study and others conducted by state wildlife-monitoring programs are the most reliable and consistent datasets currently available for long-term, regional studies of North American bats. Sampling effort across the region each year is extensive, and the long-term nature of these data provides an important historical record of population trends in these bat species. Despite the great utility of this dataset, however, it exhibited several potential sampling problems that are typical of long-term regional monitoring programs [41]. It also exhibited potential sampling problems that likely result from the cryptic nature of bats and the difficulty of monitoring them.

In this paper, we addressed several of these potential problems that would otherwise have biased our results. However, we were unable to account for other potential problems, in part due to limitations of the dataset. For instance, the survey routes in the state monitoring programs that provided data for this study were established in known hibernacula where hibernating bats could be reliably observed, and as a result easily-surveyed and consistently-used hibernacula may be overrepresented in the data. The extent and net impact of such potential sampling bias (towards over or underestimation) from the use of these particular sites for monitoring cannot be determined from our data. In addition, due to concerns about disturbance of bats during the hibernation period [12], surveys were not repeated on the same survey route in the same season (i.e., at most one survey was conducted per route per year). This aspect of the dataset limited our ability to examine interactions between temporal variables and to improve confidence estimates via modeling of detection probabilities. Also, our ability to identify key threats associated with population changes was limited by a lack of data collected concurrently on factors that may be correlated with bat counts. This limited us to using counts as an index of abundance, an approach that could affect interpretation of abundance changes if detection probability for a species changed over time. Finally, although we used a 13-year dataset, further historical data would have been beneficial to fully characterize population dynamics prior to the onset of recent declines and to more precisely identify the baseline from which to calculate cumulative declines.

Such potential sampling problems could be addressed through changes in the selection of survey routes and improvements in the survey methods employed in the monitoring programs. Questions about representativeness could be addressed through comprehensive efforts to document all hibernacula followed by implementation of random sampling of hibernacula within the entire set of hibernacula in the region. Improved survey methods could also be developed that use limited-disturbance survey methods (e.g., thermal imaging video recording) that would allow some within-season repeated measures to improve estimation of bat detectability [61]. Improved survey methods could include the use of double-observer methods for bat counts during hibernation surveys that distinguish between observer error and natural variability [49], [98], [99]. Survey efforts could also be expanded to include the recording of standardized detection covariates (e.g., hours of effort, length of survey route, and distance from observer to bat clusters) during hibernation surveys [49], [61]. This information could increase the accuracy and precision of estimates of bat abundance, and facilitate efforts to distinguish the relative importance of multiple threats driving declines. Continued monitoring efforts are also needed to improve the estimation of population dynamics over time and directly and accurately document further changes in conservation status. Other steps that could increase the accuracy and consistency of hibernation surveys are discussed in detail elsewhere (e.g., [41]).

### Modeling approach

Some disadvantages of our modeling approach were also apparent. Our results suggest that, despite our use of data from 636 surveys from 163 survey routes across four states, regional population estimates were subject to substantial uncertainties (Fig. 3). In addition, although temporal smoothing assisted with identifying long-term regional patterns in highly-variable count data, the smoothing could have had the side effect of obscuring single-year changes in population trajectories. Other factors than the observation heterogeneities we accounted for in this study, such as within- and among-year climatic variation, could also have affected bat detectability and thus estimates of population abundance. In addition, our modeling approach would need to be modified before being applied to datasets that exhibit significant deviations from the Poisson assumption such as overdispersion.

Despite these caveats, our modeling approach provided several important benefits. First, we were able to provide estimates of not only relative abundance but also uncertainty (Fig. 3; Appendix S3, S4). Both of these elements are essential to effective management decisions, but few studies of bat populations have presented estimates of uncertainty [40]. Our confidence intervals are wide in some places (Fig. 3; Appendix S4), reflecting uncertainty in point estimates due to the high spatial variability and irruptive temporal variability (P. de Valpine, T.E. Ingersoll & W. Rainey, unpublished) present in bat counts at individual hibernacula. Methods for confidence interval estimation and interpretation for time series data in GAMMs are incompletely developed, and should be the focus of future study (P. de Valpine, T.E. Ingersoll & W. Rainey, unpublished). Nonetheless, our results demonstrated strong evidence of change at the regional scale in the abundance of each species over time (Fig. 3; *Year* or smoothed *Year* terms, Appendix S3). Thus, our modeling approach reduced bias and improved interpretability of long-term regional population changes (Fig. 3), despite high short-term and local variability.

Second, our modeling approach provided estimates over time – not just before/after snapshots – while accommodating non-linearities in population abundances over time. This aspect of the modeling approach is important because there is no reason to assume, as is routine [49], that changes in population abundance over time will be linear [53]. Rather, non-linearity is especially likely to be observed in long-term studies of populations that may be responding to the combined influences of multiple environmental factors and potentially complex suites of threats. Our use of a modeling approach that accommodated non-linearity enabled us to identify time-dependent changes in the population trajectories and clarify the timing and extent of increases and declines in population abundance, allowing inference of changes in trend.

Third, trajectory estimates over time accounted for non-independent repeated-measures along sampling routes. The selection of ‘Route within location’ for *M. lucifugus* (Table 1A) but not the other species (Tables 1B, 1C, 1D) likely reflects both environmental preferences for roost sites and sample size differences. *M. lucifugus* appeared more commonly in more complex hibernacula that were surveyed with several routes. In addition, the greater amount of data for *M. lucifugus* (colony sizes as well as numbers of surveys and routes in which it was detected) enabled route-specific differences to be detected for this species in these more complex hibernacula that were surveyed with several routes. More generally, the use of the random grouping term for location enabled us to relax independence assumptions, allowing us to integrate the survey data from diverse sites – including data from the single routes that are sufficient to survey simple hibernacula and data from the multiple, non-independent routes that are needed to adequately survey complex hibernacula – in the same analysis. It also improved estimation of temporal changes in abundance by accounting for some of the detection heterogeneity associated with survey route.

Fourth, our modeling approach controlled for observation heterogeneities. Controlling for changes in within-year variation in survey date was particularly important, due to the potential of such changes to systematically bias results over time. For example, we observed a shift to later survey dates beginning near the end of the study period (Fig. 1). According to survey notes associated with hibernacula surveys, this shift was an intentional effort by managers of the monitoring programs to facilitate the detection of white-nose syndrome (which is easier to observe later in the hibernation season; T.E. Ingersoll, unpublished) after its discovery in 2007. However, such variation in survey dates (Fig. 2) could combine with systematic, within-season changes in bat abundances [24] and movements within and among hibernacula [55], [56], [57] to affect long-term, regional estimates of relative abundance (Fig. 3). Specifically, our models suggested that, if not accounted for in models, changing survey dates would have biased abundance estimates for *M. lucifugus*, *M. sodalis*, and *M. septentrionalis* (Fig. 3A, 3C, 3D; Appendix S3). Our focus on addressing observation heterogeneities explicitly addressed this potential source of bias and thereby improved the accuracy of our results.

Thus, our modeling approach proved useful in clarifying long-term, regional trajectories of populations of hibernating bat species on the basis of count data, by providing estimates of uncertainty, accommodating non-linearities, and accounting for observation heterogeneities. Such factors are often important in studies of population abundances [40], [48], [49], [50], [54], and thus our approach has wide potential applicability to other studies of wildlife populations.

## Supporting Information

Appendix S1 **Data.**
(DOC)

Appendix S2 **Sample R code for GAMMs used in this study.**
(DOC)

Appendix S3 **Tables of selected model terms for** ***Day*** **and** ***Year*** **for each bat species.**
(DOC)

Appendix S4 **Proportion of maximum expected value by year for each bat species.**
(DOC)
