# Supplementary material for: Improved Analysis of Long-Term Monitoring Data Demonstrates Marked Regional Declines of Bat Populations in the Eastern United States
Source: PLoS One. 2013 Jun 21;8(6):e65907. doi: 10.1371/journal.pone.0065907 (PMC3689752; doi:10.1371/journal.pone.0065907)
Supplement: Appendix S1 — Data. (DOC) [file pone.0065907.s001.doc]

Appendix S5. Data

M. lucifugus

| Location | Route | Day | Year | Count |
| --- | --- | --- | --- | --- |
| 1 | 1 | 0.64 | 0 | 1109 |
| 1 | 1 | 0.613333 | 0.166667 | 980 |
| 1 | 1 | 0.766667 | 0.416667 | 849 |
| 1 | 1 | 0.793333 | 0.583333 | 538 |
| 1 | 1 | 0.653333 | 0.833333 | 574 |
| 1 | 1 | 0.506667 | 0.916667 | 306 |
| 2 | 2 | 0.613333 | 0.333333 | 297 |
| 2 | 2 | 0.673333 | 0.916667 | 339 |
| 2 | 2 | 0.673333 | 1 | 418 |
| 3 | 3 | 0.613333 | 0.333333 | 17 |
| 3 | 3 | 0.68 | 0.833333 | 4 |
| 4 | 4 | 0.766667 | 0.25 | 861 |
| 4 | 4 | 0.686667 | 0.333333 | 937 |
| 4 | 4 | 0.7 | 0.416667 | 1465 |
| 4 | 4 | 0.773333 | 0.583333 | 492 |
| 4 | 4 | 0.826667 | 0.833333 | 672 |
| 4 | 4 | 0.813333 | 0.916667 | 392 |
| 5 | 5 | 0.52 | 0.166667 | 21 |
| 5 | 5 | 0.66 | 0.5 | 0 |
| 6 | 6 | 0.58 | 0 | 133 |
| 6 | 6 | 0.486667 | 0.166667 | 74 |
| 6 | 6 | 0.346667 | 0.5 | 0 |
| 7 | 7 | 0.78 | 0 | 1 |
| 7 | 7 | 0.62 | 0.083333 | 2 |
| 8 | 8 | 0.713333 | 0 | 309 |
| 8 | 8 | 0.826667 | 0.416667 | 256 |
| 8 | 8 | 0.84 | 0.583333 | 352 |
| 8 | 8 | 0.873333 | 0.75 | 278 |
| 8 | 8 | 0.953333 | 0.833333 | 157 |
| 8 | 8 | 0.913333 | 0.916667 | 115 |
| 8 | 8 | 0.526667 | 1 | 84 |
| 9 | 9 | 0.68 | 0.583333 | 1 |
| 9 | 9 | 0.446667 | 0.75 | 3 |
| 9 | 9 | 0.666667 | 0.833333 | 7 |
| 9 | 9 | 0.713333 | 0.916667 | 0 |
| 9 | 9 | 0.64 | 1 | 3 |
| 10 | 10 | 0.033333 | 0.583333 | 1511 |
| 10 | 10 | 0.92 | 0.75 | 1 |
| 10 | 10 | 0.64 | 1 | 1 |
| 11 | 11 | 0.493333 | 0 | 4 |
| 11 | 11 | 0.546667 | 0.083333 | 0 |
| 11 | 11 | 0.56 | 0.166667 | 0 |
| 11 | 11 | 0.646667 | 0.25 | 5 |
| 11 | 11 | 0.46 | 0.333333 | 0 |
| 11 | 11 | 0.64 | 0.416667 | 3 |
| 11 | 11 | 0.533333 | 0.5 | 3 |
| 11 | 11 | 0.473333 | 0.666667 | 9 |
| 11 | 11 | 0.56 | 0.833333 | 0 |
| 12 | 12 | 0.6 | 0 | 23125 |
| 12 | 12 | 0.586667 | 0.333333 | 17399 |
| 12 | 12 | 0.453333 | 0.916667 | 1669 |
| 12 | 12 | 0.586667 | 1 | 1450 |
| 13 | 13 | 0.76 | 0.583333 | 37 |
| 13 | 13 | 0.946667 | 0.75 | 4 |
| 14 | 14 | 0.533333 | 0 | 788 |
| 14 | 14 | 0.346667 | 0.166667 | 354 |
| 14 | 14 | 0.473333 | 0.333333 | 769 |
| 14 | 14 | 0.3 | 0.5 | 900 |
| 14 | 14 | 0.52 | 0.666667 | 1430 |
| 14 | 14 | 0.346667 | 0.833333 | 1328 |
| 14 | 14 | 0.6 | 0.916667 | 147 |
| 14 | 14 | 0.993333 | 1 | 691 |
| 15 | 15 | 0.8 | 0.583333 | 107 |
| 15 | 15 | 0.58 | 0.916667 | 10 |
| 16 | 16 | 0.553333 | 0.083333 | 114 |
| 16 | 16 | 0.58 | 0.666667 | 58 |
| 17 | 17 | 0.546667 | 0.083333 | 13 |
| 17 | 17 | 0.56 | 0.166667 | 15 |
| 17 | 17 | 0.46 | 0.333333 | 10 |
| 17 | 17 | 0.426667 | 0.416667 | 26 |
| 17 | 17 | 0.533333 | 0.5 | 19 |
| 17 | 17 | 0.473333 | 0.666667 | 45 |
| 17 | 17 | 0.98 | 0.75 | 56 |
| 17 | 17 | 0.56 | 0.833333 | 64 |
| 17 | 17 | 0.826667 | 0.916667 | 92 |
| 17 | 17 | 0.473333 | 1 | 80 |
| 18 | 18 | 0.933333 | 0.75 | 975 |
| 18 | 18 | 0.826667 | 0.916667 | 161 |
| 18 | 18 | 0.96 | 1 | 194 |
| 19 | 19 | 0.62 | 0.666667 | 3 |
| 19 | 19 | 0.786667 | 0.916667 | 0 |
| 19 | 19 | 0.826667 | 1 | 3 |
| 20 | 20 | 0.686667 | 0 | 442 |
| 20 | 20 | 0.453333 | 0.083333 | 4 |
| 20 | 20 | 0.493333 | 0.166667 | 10 |
| 20 | 20 | 0.766667 | 0.25 | 745 |
| 20 | 20 | 0.413333 | 0.583333 | 8 |
| 20 | 20 | 0.773333 | 0.833333 | 36 |
| 20 | 20 | 0.86 | 0.916667 | 80 |
| 21 | 21 | 0.62 | 0.666667 | 19 |
| 21 | 21 | 0.786667 | 0.916667 | 102 |
| 21 | 21 | 0.913333 | 1 | 21 |
| 22 | 22 | 0.633333 | 0 | 13180 |
| 22 | 22 | 0.746667 | 0.166667 | 12839 |
| 22 | 22 | 0.753333 | 0.333333 | 13502 |
| 22 | 22 | 0.76 | 0.5 | 10875 |
| 22 | 22 | 0.746667 | 0.666667 | 6155 |
| 23 | 23 | 0.58 | 0.083333 | 41 |
| 23 | 23 | 0.84 | 0.25 | 0 |
| 24 | 24 | 0.68 | 0.083333 | 4 |
| 24 | 24 | 0.586667 | 0.5 | 2 |
| 24 | 24 | 0.606667 | 0.583333 | 33 |
| 24 | 24 | 0.693333 | 0.666667 | 0 |
| 24 | 24 | 0.426667 | 0.75 | 4 |
| 25 | 25 | 0.62 | 0.583333 | 574 |
| 25 | 25 | 0.433333 | 0.75 | 308 |
| 25 | 25 | 0.64 | 0.916667 | 172 |
| 26 | 26 | 0.58 | 0 | 34 |
| 26 | 26 | 0.486667 | 0.166667 | 81 |
| 26 | 26 | 0.866667 | 0.416667 | 0 |
| 26 | 26 | 0.346667 | 0.5 | 43 |
| 26 | 26 | 0.3 | 0.666667 | 39 |
| 26 | 26 | 0.466667 | 0.833333 | 19 |
| 27 | 27 | 0.86 | 0.083333 | 149 |
| 27 | 27 | 0.606667 | 0.333333 | 231 |
| 27 | 27 | 0.673333 | 0.5 | 146 |
| 27 | 27 | 0.806667 | 0.666667 | 209 |
| 27 | 27 | 0.64 | 0.833333 | 318 |
| 27 | 27 | 0.713333 | 1 | 17 |
| 28 | 28 | 0.586667 | 0 | 0 |
| 28 | 28 | 0.7 | 0.166667 | 3 |
| 28 | 28 | 0.553333 | 0.333333 | 2 |
| 28 | 28 | 0.713333 | 0.5 | 6 |
| 28 | 28 | 0.746667 | 0.666667 | 21 |
| 28 | 28 | 0.826667 | 0.833333 | 92 |
| 29 | 29 | 0.726667 | 0.083333 | 1261 |
| 29 | 29 | 0.66 | 0.583333 | 1035 |
| 29 | 29 | 0.553333 | 0.833333 | 796 |
| 29 | 29 | 0.92 | 1 | 8 |
| 30 | 30 | 0.633333 | 0.666667 | 370 |
| 30 | 30 | 0.726667 | 1 | 509 |
| 31 | 31 | 0.78 | 0.083333 | 193 |
| 31 | 31 | 0.653333 | 0.25 | 99 |
| 31 | 31 | 0.926667 | 0.5 | 309 |
| 31 | 31 | 0.906667 | 0.916667 | 87 |
| 32 | 32 | 0.713333 | 0.083333 | 26300 |
| 32 | 32 | 0.666667 | 0.25 | 26645 |
| 32 | 32 | 0.726667 | 0.416667 | 30653 |
| 32 | 32 | 0.7 | 0.75 | 17616 |
| 33 | 33 | 0.653333 | 0.75 | 1411 |
| 33 | 33 | 0.626667 | 0.833333 | 1822 |
| 33 | 33 | -0.01333 | 0.916667 | 2000 |
| 34 | 34 | 0.533333 | 0.083333 | 51 |
| 34 | 34 | 0.566667 | 0.25 | 46 |
| 34 | 34 | 0.513333 | 0.416667 | 57 |
| 34 | 34 | 0.493333 | 0.583333 | 69 |
| 34 | 34 | 0.6 | 0.75 | 61 |
| 35 | 35 | 0.9 | 0.916667 | 0 |
| 35 | 35 | 0.773333 | 1 | 0 |
| 36 | 36 | 0.693333 | 0.166667 | 1 |
| 36 | 36 | 0.606667 | 0.583333 | 0 |
| 37 | 37 | 0.746667 | 0.25 | 5783 |
| 37 | 37 | 0.78 | 0.416667 | 4982 |
| 37 | 37 | 0.8 | 0.583333 | 7356 |
| 37 | 37 | 0.646667 | 0.75 | 8355 |
| 37 | 37 | 0.86 | 0.833333 | 5648 |
| 37 | 37 | -0.02 | 0.916667 | 10425 |
| 37 | 37 | 0.906667 | 1 | 8048 |
| 38 | 38 | 0.66 | 0 | 217 |
| 38 | 38 | 0.78 | 0.166667 | 395 |
| 38 | 38 | 0.693333 | 0.833333 | 1469 |
| 39 | 39 | 0.593333 | 0.583333 | 200 |
| 39 | 39 | 0.906667 | 1 | 273 |
| 40 | 40 | 0.48 | 0.166667 | 41 |
| 40 | 40 | 0.72 | 0.916667 | 80 |
| 41 | 41 | 0.68 | 0.083333 | 407 |
| 41 | 41 | 0.786667 | 0.833333 | 14 |
| 41 | 41 | 0.946667 | 0.916667 | 0 |
| 42 | 42 | 0.573333 | 0.166667 | 33 |
| 42 | 42 | 0.58 | 0.416667 | 59 |
| 42 | 42 | 0.493333 | 0.583333 | 27 |
| 43 | 43 | 0.66 | 0.083333 | 279 |
| 43 | 43 | 0.6 | 0.25 | 261 |
| 43 | 43 | 0.493333 | 0.416667 | 251 |
| 43 | 43 | 0.586667 | 0.583333 | 272 |
| 43 | 43 | 0.566667 | 0.75 | 352 |
| 44 | 44 | 0.566667 | 0.416667 | 1102 |
| 44 | 44 | 0.926667 | 0.833333 | 22 |
| 45 | 45 | 0.806667 | 0.75 | 93 |
| 45 | 45 | 0.72 | 0.833333 | 56 |
| 45 | 45 | 0.913333 | 0.916667 | 37 |
| 45 | 45 | 0.586667 | 1 | 40 |
| 46 | 46 | 0.54 | 0.083333 | 5 |
| 46 | 46 | 0.84 | 0.25 | 3 |
| 46 | 46 | 0.606667 | 0.416667 | 2 |
| 46 | 46 | 0.533333 | 0.583333 | 1 |
| 47 | 47 | 0.606667 | 0.333333 | 37436 |
| 47 | 47 | 0.666667 | 0.5 | 18190 |
| 47 | 47 | 0.693333 | 0.75 | 0 |
| 48 | 48 | 0.72 | 0.083333 | 12 |
| 48 | 48 | 0.753333 | 0.25 | 28 |
| 48 | 48 | 0.726667 | 0.416667 | 31 |
| 48 | 48 | 0.326667 | 0.583333 | 16 |
| 48 | 48 | 0.66 | 0.75 | 28 |
| 49 | 49 | 0.54 | 0.416667 | 3 |
| 49 | 49 | 0.913333 | 0.5 | 0 |
| 50 | 50 | 0.76 | 0 | 241 |
| 50 | 50 | 0.753333 | 0.166667 | 241 |
| 50 | 50 | 0.78 | 0.333333 | 151 |
| 50 | 50 | 0.76 | 0.5 | 13 |
| 50 | 50 | 0.793333 | 0.666667 | 37 |
| 50 | 50 | 0.766667 | 0.833333 | 22 |
| 50 | 50 | 0.726667 | 0.916667 | 10 |
| 50 | 50 | 0.8 | 1 | 12 |
| 51 | 51 | 0.76 | 0 | 5 |
| 51 | 51 | 0.753333 | 0.166667 | 10 |
| 51 | 51 | 0.78 | 0.333333 | 0 |
| 52 | 52 | 0.72 | 0.083333 | 2105 |
| 52 | 52 | 0.746667 | 0.25 | 2344 |
| 52 | 52 | 0.733333 | 0.416667 | 2572 |
| 52 | 52 | 0.713333 | 0.583333 | 2294 |
| 52 | 52 | 0.693333 | 0.75 | 3622 |
| 52 | 52 | 0.773333 | 0.916667 | 2197 |
| 53 | 53 | 0.853333 | 0.916667 | 0 |
| 53 | 53 | 0.773333 | 1 | 4 |
| 54 | 54 | 0.613333 | 0.166667 | 2 |
| 54 | 54 | 0.533333 | 0.583333 | 0 |
| 55 | 55 | 0.666667 | 0 | 7793 |
| 55 | 55 | 0.9 | 0.166667 | 2012 |
| 55 | 55 | 0.793333 | 0.333333 | 9868 |
| 55 | 55 | 0.82 | 0.5 | 15374 |
| 55 | 55 | 0.893333 | 0.666667 | 7258 |
| 55 | 55 | 0.8 | 0.75 | 1443 |
| 55 | 55 | 0.773333 | 0.833333 | 1000 |
| 55 | 55 | 0.826667 | 0.916667 | 1198 |
| 55 | 55 | 0.733333 | 1 | 1496 |
| 56 | 56 | 0.753333 | 0.083333 | 0 |
| 56 | 56 | 0.553333 | 0.333333 | 49 |
| 56 | 56 | 0.953333 | 1 | 13 |
| 57 | 57 | 0.54 | 0.666667 | 267 |
| 57 | 57 | 0.673333 | 0.75 | 41 |
| 58 | 58 | 0.906667 | 0.583333 | 2922 |
| 58 | 58 | 0.913333 | 0.833333 | 1218 |
| 59 | 59 | 0.773333 | 0.083333 | 6 |
| 59 | 59 | 0.673333 | 0.916667 | 0 |
| 60 | 60 | 0.773333 | 0.083333 | 38 |
| 60 | 60 | 0.673333 | 0.916667 | 0 |
| 61 | 61 | 0.773333 | 0.166667 | 23057 |
| 61 | 61 | 0.82 | 0.5 | 21357 |
| 61 | 61 | 0.773333 | 0.666667 | 26665 |
| 61 | 61 | 0.68 | 0.916667 | 19500 |
| 61 | 62 | 0.793333 | 0 | 14710 |
| 61 | 62 | 0.773333 | 0.166667 | 32226 |
| 61 | 63 | 0.793333 | 0 | 2064 |
| 61 | 63 | 0.773333 | 0.166667 | 1914 |
| 61 | 63 | 0.82 | 0.5 | 2616 |
| 61 | 63 | 0.773333 | 0.666667 | 3319 |
| 61 | 63 | 0.68 | 0.916667 | 1800 |
| 61 | 64 | 0.793333 | 0 | 1070 |
| 61 | 64 | 0.773333 | 0.166667 | 205 |
| 61 | 64 | 0.82 | 0.5 | 5254 |
| 61 | 64 | 0.773333 | 0.666667 | 2549 |
| 61 | 64 | 0.68 | 0.916667 | 2002 |
| 61 | 65 | 0.773333 | 0.666667 | 40179 |
| 61 | 65 | 0.68 | 0.916667 | 14253 |
| 61 | 66 | 0.82 | 0.5 | 18128 |
| 61 | 66 | 0.773333 | 0.666667 | 22134 |
| 62 | 67 | 0.606667 | 0.166667 | 19 |
| 62 | 67 | 0.806667 | 0.333333 | 37 |
| 63 | 68 | 0.433333 | 0 | 893 |
| 63 | 68 | 0.66 | 0.166667 | 804 |
| 63 | 68 | 0.66 | 0.333333 | 697 |
| 63 | 68 | 0.76 | 0.5 | 1013 |
| 63 | 68 | 0.626667 | 0.666667 | 720 |
| 63 | 68 | 0.6 | 0.833333 | 795 |
| 63 | 68 | 0.493333 | 1 | 137 |
| 64 | 69 | 0.92 | 0 | 256 |
| 64 | 69 | 0.726667 | 0.083333 | 87 |
| 64 | 69 | 0.733333 | 0.416667 | 256 |
| 64 | 69 | 0.706667 | 0.583333 | 411 |
| 64 | 69 | 0.74 | 0.75 | 88 |
| 64 | 69 | 0.773333 | 0.833333 | 48 |
| 65 | 70 | 0.693333 | 0.833333 | 646 |
| 65 | 70 | 0.673333 | 0.916667 | 450 |
| 66 | 71 | 0.88 | 0.416667 | 1011 |
| 66 | 71 | 0.873333 | 0.5 | 1213 |
| 66 | 71 | 0.746667 | 0.583333 | 1108 |
| 66 | 71 | 0.74 | 0.666667 | 142 |
| 66 | 71 | 0.446667 | 0.75 | 62 |
| 66 | 71 | 0.7 | 0.833333 | 27 |
| 66 | 71 | 0.646667 | 0.916667 | 37 |
| 66 | 71 | 0.64 | 1 | 29 |
| 67 | 72 | 0.646667 | 0.916667 | 0 |
| 67 | 72 | 0.64 | 1 | 4 |
| 68 | 73 | 0.646667 | 0.916667 | 0 |
| 68 | 73 | 0.64 | 1 | 2 |
| 69 | 74 | 0.68 | 0.583333 | 0 |
| 69 | 74 | 0.52 | 0.75 | 1 |
| 69 | 74 | 0.713333 | 0.916667 | 1 |
| 70 | 75 | 0.44 | 0.083333 | 145 |
| 70 | 75 | 0.52 | 0.25 | 91 |
| 70 | 75 | 0.553333 | 0.416667 | 117 |
| 70 | 75 | 0.533333 | 0.583333 | 160 |
| 70 | 75 | 0.573333 | 0.75 | 272 |
| 71 | 76 | 0.62 | 0 | 469 |
| 71 | 76 | 0.626667 | 0.166667 | 262 |
| 71 | 76 | 0.78 | 0.333333 | 1346 |
| 71 | 76 | 0.813333 | 0.916667 | 1435 |
| 71 | 76 | 0.713333 | 1 | 573 |
| 72 | 77 | 0.733333 | 0 | 7 |
| 72 | 77 | 0.74 | 1 | 5 |
| 73 | 78 | 0.733333 | 0 | 50 |
| 73 | 78 | 0.74 | 1 | 59 |
| 74 | 79 | 0.533333 | 0.083333 | 0 |
| 74 | 79 | 0.566667 | 0.25 | 0 |
| 74 | 79 | 0.513333 | 0.416667 | 0 |
| 74 | 79 | 0.493333 | 0.583333 | 1 |
| 75 | 80 | 0.68 | 0.666667 | 5 |
| 75 | 80 | 0.94 | 0.75 | 1 |
| 76 | 81 | 0.566667 | 0.833333 | 17 |
| 76 | 81 | 0.806667 | 1 | 4 |
| 77 | 82 | 0.613333 | 0.333333 | 3 |
| 77 | 82 | 0.566667 | 0.666667 | 17 |
| 77 | 82 | 0.78 | 1 | 25 |
| 78 | 83 | 0.266667 | 0.25 | 1820 |
| 78 | 83 | 0.88 | 0.75 | 363 |
| 78 | 83 | 0.72 | 0.833333 | 179 |
| 78 | 83 | 0.606667 | 1 | 354 |
| 79 | 84 | 0.88 | 0.25 | 744 |
| 79 | 84 | 0.593333 | 0.5 | 532 |
| 79 | 84 | 0.9 | 0.75 | 1059 |
| 80 | 85 | 0.72 | 0 | 4 |
| 80 | 85 | 0.666667 | 0.083333 | 0 |
| 80 | 85 | 0.66 | 0.25 | 0 |
| 80 | 85 | 0.633333 | 0.583333 | 0 |
| 81 | 86 | 0.58 | 0 | 6489 |
| 81 | 86 | 0.526667 | 0.166667 | 4601 |
| 81 | 86 | 0.613333 | 0.333333 | 4135 |
| 81 | 86 | 0.58 | 0.416667 | 3181 |
| 81 | 86 | 0.62 | 0.583333 | 3174 |
| 81 | 86 | 0.793333 | 0.75 | 2851 |
| 81 | 86 | 0.586667 | 0.833333 | 3765 |
| 81 | 86 | 0.866667 | 1 | 2527 |
| 82 | 87 | 0.593333 | 0 | 825 |
| 82 | 87 | 0.7 | 0.166667 | 1160 |
| 82 | 87 | 0.673333 | 0.416667 | 937 |
| 82 | 87 | 0.666667 | 0.583333 | 1038 |
| 82 | 87 | 0.56 | 0.833333 | 909 |
| 83 | 88 | 0.826667 | 0.583333 | 5 |
| 83 | 88 | 0.9 | 0.75 | 21 |
| 84 | 89 | 0.906667 | 0.916667 | 0 |
| 84 | 89 | 0.813333 | 1 | 0 |
| 85 | 90 | 0.606667 | 0.666667 | 5 |
| 85 | 90 | 1 | 1 | 4 |
| 86 | 91 | 0.833333 | 0.25 | 5 |
| 86 | 91 | 0.633333 | 0.5 | 0 |
| 87 | 92 | 0.633333 | 0.083333 | 616 |
| 87 | 92 | 0.686667 | 0.25 | 898 |
| 87 | 92 | 0.646667 | 0.416667 | 1190 |
| 87 | 92 | 0.666667 | 0.583333 | 947 |
| 87 | 92 | 0.64 | 0.75 | 427 |
| 87 | 92 | 0.673333 | 1 | 411 |
| 88 | 93 | 0.96 | 0.75 | 434 |
| 88 | 93 | 0.926667 | 0.833333 | 117 |
| 88 | 93 | 0.74 | 0.916667 | 39 |
| 89 | 94 | 0.613333 | 0.333333 | 84 |
| 89 | 94 | 0.68 | 0.5 | 172 |
| 89 | 94 | 0.853333 | 0.666667 | 111 |
| 90 | 95 | 0.433333 | 0 | 733 |
| 90 | 95 | 0.66 | 0.166667 | 514 |
| 90 | 95 | 0.66 | 0.333333 | 224 |
| 90 | 95 | 0.72 | 0.5 | 313 |
| 90 | 95 | 0.606667 | 0.666667 | 630 |
| 90 | 95 | 0.86 | 0.833333 | 832 |
| 90 | 95 | 0.753333 | 1 | 37 |
| 91 | 96 | 0.666667 | 0.916667 | 0 |
| 91 | 96 | 0.773333 | 1 | 1 |
| 92 | 97 | 0.626667 | 0 | 9 |
| 92 | 97 | 0.586667 | 0.083333 | 17 |
| 92 | 97 | 0.82 | 0.25 | 12 |
| 92 | 97 | 0.553333 | 0.333333 | 9 |
| 92 | 97 | 0.626667 | 0.5 | 7 |
| 92 | 97 | 0.906667 | 0.833333 | 8 |
| 92 | 97 | 0.873333 | 1 | 0 |
| 93 | 98 | 0.82 | 0.25 | 0 |
| 93 | 98 | 0.626667 | 0.5 | 0 |
| 94 | 99 | 0.413333 | 0.5 | 444 |
| 94 | 99 | 0.68 | 0.666667 | 402 |
| 94 | 99 | 0.726667 | 0.916667 | 325 |
| 94 | 99 | 0.76 | 1 | 532 |
| 95 | 100 | 0.653333 | 0 | 0 |
| 95 | 100 | 0.606667 | 0.833333 | 8 |
| 96 | 101 | 0.633333 | 0.083333 | 224 |
| 96 | 101 | 0.773333 | 0.416667 | 104 |
| 96 | 101 | 0.8 | 0.583333 | 91 |
| 96 | 101 | 0.646667 | 0.75 | 53 |
| 96 | 101 | 0.346667 | 0.833333 | 82 |
| 96 | 101 | 0.9 | 1 | 84 |
| 97 | 102 | 0.713333 | 0.083333 | 1512 |
| 97 | 102 | 0.753333 | 0.25 | 1579 |
| 97 | 102 | 0.806667 | 0.416667 | 1356 |
| 97 | 102 | 0.7 | 0.583333 | 1690 |
| 97 | 102 | 0.686667 | 0.75 | 1947 |
| 97 | 102 | 0.766667 | 1 | 1060 |
| 98 | 103 | 0.726667 | 0.083333 | 343 |
| 98 | 103 | 0.746667 | 0.25 | 530 |
| 98 | 103 | 0.733333 | 0.416667 | 388 |
| 98 | 103 | 0.706667 | 0.583333 | 625 |
| 98 | 103 | 0.686667 | 0.75 | 802 |
| 98 | 103 | 0.853333 | 0.916667 | 814 |
| 98 | 103 | 0.846667 | 1 | 118 |
| 99 | 104 | 0.593333 | 0 | 4 |
| 99 | 104 | 0.533333 | 0.166667 | 30 |
| 99 | 104 | 0.506667 | 0.333333 | 3 |
| 99 | 104 | 0.313333 | 0.5 | 0 |
| 99 | 104 | 0.533333 | 0.666667 | 8 |
| 99 | 104 | 0.686667 | 0.833333 | 8 |
| 100 | 105 | 0.586667 | 0.583333 | 792 |
| 100 | 105 | 0.766667 | 0.666667 | 616 |
| 101 | 106 | 0.713333 | 0.25 | 0 |
| 101 | 106 | 0.753333 | 0.333333 | 2 |
| 101 | 106 | 0.54 | 0.583333 | 0 |
| 102 | 107 | 0.713333 | 0.083333 | 1763 |
| 102 | 107 | 0.74 | 0.25 | 1973 |
| 102 | 107 | 0.726667 | 0.416667 | 1913 |
| 102 | 107 | 0.706667 | 0.583333 | 2223 |
| 102 | 107 | 0.673333 | 0.75 | 1823 |
| 103 | 108 | 0.533333 | 0 | 2 |
| 103 | 108 | 0.833333 | 0.25 | 5 |
| 103 | 108 | 0.693333 | 0.333333 | 14 |
| 103 | 108 | 0.64 | 0.5 | 0 |
| 104 | 109 | 0.766667 | 0.166667 | 0 |
| 104 | 109 | 0.893333 | 0.75 | 0 |
| 104 | 109 | 0.686667 | 0.833333 | 0 |
| 105 | 110 | 0.813333 | 0.916667 | 19 |
| 105 | 110 | 0.726667 | 1 | 12 |
| 106 | 111 | 0.62 | 0.666667 | 5 |
| 106 | 111 | 0.826667 | 0.916667 | 6 |
| 107 | 112 | 0.573333 | 0.083333 | 256 |
| 107 | 112 | 0.76 | 0.25 | 197 |
| 107 | 112 | 0.706667 | 0.5 | 204 |
| 107 | 112 | 0.753333 | 0.666667 | 157 |
| 107 | 112 | 0.866667 | 0.833333 | 131 |
| 107 | 112 | 0.94 | 1 | 48 |
| 108 | 113 | 0.726667 | 0 | 2103 |
| 108 | 113 | 0.66 | 0.083333 | 2123 |
| 108 | 113 | 0.653333 | 0.25 | 1636 |
| 108 | 113 | 0.56 | 0.416667 | 1602 |
| 108 | 113 | 0.673333 | 0.583333 | 1434 |
| 108 | 113 | 0.513333 | 0.666667 | 1275 |
| 108 | 113 | 0.5 | 0.75 | 1191 |
| 108 | 113 | 1 | 0.916667 | 1352 |
| 109 | 114 | 0.72 | 0 | 94 |
| 109 | 114 | 0.673333 | 0.583333 | 88 |
| 110 | 115 | 0.806667 | 0.416667 | 7 |
| 110 | 115 | 0.5 | 1 | 6 |
| 111 | 116 | 0.573333 | 0 | 518 |
| 111 | 116 | 0.66 | 0.166667 | 426 |
| 111 | 116 | 0.746667 | 0.333333 | 431 |
| 111 | 116 | 0.826667 | 0.5 | 206 |
| 112 | 117 | 0.793333 | 0 | 953 |
| 112 | 117 | 0.566667 | 0.75 | 28 |
| 112 | 117 | 0.84 | 0.833333 | 34 |
| 112 | 117 | 0.88 | 0.916667 | 22 |
| 113 | 118 | 0.606667 | 0 | 0 |
| 113 | 118 | 0.673333 | 0.166667 | 1 |
| 113 | 118 | 0.7 | 0.333333 | 0 |
| 113 | 118 | 0.626667 | 0.5 | 1 |
| 113 | 118 | 0.713333 | 0.666667 | 0 |
| 113 | 118 | 0.733333 | 0.833333 | 1 |
| 113 | 118 | 0.813333 | 0.916667 | 17 |
| 113 | 118 | 0.826667 | 1 | 2 |
| 114 | 119 | 0.62 | 0 | 2352 |
| 114 | 119 | 0.74 | 0.166667 | 2442 |
| 114 | 119 | 0.546667 | 0.333333 | 1544 |
| 114 | 119 | 0.813333 | 0.5 | 1903 |
| 114 | 119 | 0.673333 | 0.666667 | 1262 |
| 114 | 119 | 0.753333 | 0.833333 | 747 |
| 115 | 120 | 0.673333 | 0 | 756 |
| 115 | 120 | 0.613333 | 0.25 | 645 |
| 115 | 120 | 0.6 | 0.416667 | 729 |
| 115 | 120 | 0.766667 | 0.583333 | 767 |
| 115 | 120 | 0.706667 | 0.75 | 215 |
| 116 | 121 | 0.626667 | 0 | 3 |
| 116 | 121 | 0.586667 | 0.083333 | 109 |
| 116 | 121 | 0.626667 | 0.5 | 2092 |
| 116 | 121 | 0.76 | 0.666667 | 1802 |
| 116 | 121 | 0.74 | 0.833333 | 2348 |
| 116 | 121 | 0.96 | 0.916667 | 1495 |
| 117 | 122 | 0.666667 | 0.166667 | 0 |
| 117 | 122 | 0.953333 | 0.333333 | 0 |
| 117 | 122 | 0.893333 | 0.75 | 0 |
| 117 | 122 | 0.986667 | 1 | 1 |
| 118 | 123 | 0.426667 | 0 | 53 |
| 118 | 123 | 0.666667 | 0.166667 | 40 |
| 118 | 123 | 0.546667 | 0.333333 | 58 |
| 118 | 123 | 0.76 | 0.5 | 36 |
| 118 | 123 | 0.606667 | 0.666667 | 45 |
| 118 | 123 | 0.766667 | 0.833333 | 56 |
| 118 | 123 | 0.82 | 0.916667 | 27 |
| 119 | 124 | 0.633333 | 0.083333 | 1253 |
| 119 | 124 | 0.686667 | 0.25 | 1309 |
| 119 | 124 | 0.646667 | 0.416667 | 1516 |
| 119 | 124 | 0.666667 | 0.583333 | 1726 |
| 119 | 124 | 0.64 | 0.75 | 2107 |
| 119 | 124 | 0.733333 | 0.916667 | 1307 |
| 119 | 124 | 0.813333 | 1 | 330 |
| 120 | 125 | 0.653333 | 0.333333 | 1481 |
| 120 | 125 | 0.673333 | 0.5 | 1031 |
| 120 | 125 | 0.66 | 0.666667 | 756 |
| 120 | 125 | 0.646667 | 0.833333 | 397 |
| 120 | 125 | 0.34 | 1 | 162 |
| 121 | 126 | 0.673333 | 0.5 | 891 |
| 121 | 126 | 0.646667 | 0.833333 | 392 |
| 121 | 126 | 0.493333 | 0.916667 | 227 |
| 122 | 127 | 0.846667 | 0.5 | 100 |
| 122 | 127 | 0.686667 | 0.75 | 12 |
| 122 | 127 | 0.813333 | 0.833333 | 3 |
| 122 | 127 | 0.853333 | 0.916667 | 0 |
| 122 | 127 | 0.706667 | 1 | 1 |
| 123 | 128 | 0.546667 | 0 | 1602 |
| 123 | 128 | 0.52 | 0.166667 | 1403 |
| 123 | 128 | 0.733333 | 0.416667 | 1035 |
| 123 | 128 | 0.653333 | 0.583333 | 1577 |
| 124 | 129 | 0.506667 | 0 | 154 |
| 124 | 129 | 0.626667 | 0.166667 | 405 |
| 124 | 129 | 0.686667 | 0.416667 | 422 |
| 124 | 129 | 0.633333 | 0.583333 | 562 |
| 124 | 129 | 0.786667 | 0.75 | 754 |
| 125 | 130 | 0.54 | 0.083333 | 1 |
| 125 | 130 | 0.833333 | 0.25 | 0 |
| 126 | 131 | 0.553333 | 0.166667 | 0 |
| 126 | 131 | 0.506667 | 0.833333 | 16 |
| 127 | 132 | 0.666667 | 0.083333 | 126 |
| 127 | 132 | 0.506667 | 0.833333 | 5 |
| 128 | 133 | 0.64 | 0.666667 | 10 |
| 128 | 133 | 0.68 | 1 | 246 |
| 129 | 134 | 0.653333 | 0 | 160 |
| 129 | 134 | 0.68 | 0.166667 | 113 |
| 129 | 134 | 0.666667 | 0.333333 | 119 |
| 129 | 134 | 0.693333 | 0.5 | 135 |
| 129 | 134 | 0.68 | 0.583333 | 0 |
| 129 | 134 | 0.673333 | 0.666667 | 142 |
| 129 | 134 | 0.606667 | 0.833333 | 112 |
| 129 | 134 | 0.82 | 0.916667 | 28 |
| 129 | 134 | 0.8 | 1 | 8 |
| 131 | 136 | 0.5 | 0.083333 | 735 |
| 130 | 135 | 0.62 | 0.25 | 899 |
| 131 | 136 | 0.586667 | 0.416667 | 553 |
| 131 | 136 | 0.486667 | 0.583333 | 605 |
| 131 | 136 | 0.613333 | 0.75 | 464 |
| 132 | 137 | 0.54 | 0 | 5251 |
| 132 | 137 | 0.793333 | 0.166667 | 5963 |
| 132 | 137 | 0.64 | 0.333333 | 5074 |
| 132 | 137 | 0.68 | 0.5 | 2234 |
| 132 | 137 | 0.666667 | 0.666667 | 2008 |
| 132 | 137 | 0.673333 | 0.833333 | 1024 |
| 133 | 138 | 0.633333 | 0.083333 | 17 |
| 133 | 138 | 0.686667 | 0.25 | 0 |
| 133 | 138 | 0.646667 | 0.416667 | 1 |
| 133 | 138 | 0.666667 | 0.583333 | 21 |
| 133 | 138 | 0.64 | 0.75 | 8 |
| 133 | 138 | 0.673333 | 1 | 5 |
| 134 | 139 | 0.953333 | 0 | 87401 |
| 134 | 139 | 0.633333 | 0.833333 | 10251 |
| 134 | 139 | 0.873333 | 0.916667 | 16673 |
| 135 | 140 | 1 | 0.916667 | 640 |
| 135 | 140 | 0.98 | 1 | 574 |
| 136 | 141 | 0.853333 | 0.25 | 144 |
| 136 | 141 | 0.62 | 0.666667 | 54 |
| 136 | 141 | 0.5 | 0.916667 | 1 |
| 136 | 141 | 0.92 | 1 | 1 |
| 137 | 142 | 0.853333 | 0.25 | 531 |
| 137 | 142 | 0.58 | 0.833333 | 212 |
| 137 | 142 | 0.92 | 0.916667 | 33 |
| 138 | 143 | 0.853333 | 0.25 | 1 |
| 138 | 143 | 0.293333 | 0.75 | 38 |
| 138 | 143 | 0.593333 | 0.916667 | 35 |
| 139 | 144 | 0.766667 | 0.666667 | 485 |
| 139 | 144 | 0.92 | 1 | 734 |
| 140 | 145 | 0.666667 | 0 | 2579 |
| 140 | 145 | 0.76 | 0.166667 | 2472 |
| 140 | 145 | 0.78 | 0.333333 | 1799 |
| 140 | 145 | 0.753333 | 0.583333 | 2164 |
| 140 | 145 | 0.78 | 0.75 | 1454 |
| 140 | 145 | 0.446667 | 0.916667 | 1630 |
| 140 | 145 | 0.686667 | 1 | 1232 |

***P. subflavus***

| Location | Route | Day | Year | Count |
| --- | --- | --- | --- | --- |
| 1 | 1 | 0.64 | 0 | 103 |
| 1 | 1 | 0.613333 | 0.166667 | 96 |
| 1 | 1 | 0.766667 | 0.416667 | 24 |
| 1 | 1 | 0.793333 | 0.583333 | 51 |
| 1 | 1 | 0.653333 | 0.833333 | 76 |
| 1 | 1 | 0.506667 | 0.916667 | 39 |
| 2 | 2 | 0.613333 | 0.333333 | 464 |
| 2 | 2 | 0.673333 | 0.916667 | 102 |
| 2 | 2 | 0.673333 | 1 | 200 |
| 3 | 3 | 0.613333 | 0.333333 | 289 |
| 3 | 3 | 0.68 | 0.833333 | 4 |
| 4 | 4 | 0.766667 | 0.25 | 127 |
| 4 | 4 | 0.686667 | 0.333333 | 148 |
| 4 | 4 | 0.7 | 0.416667 | 250 |
| 4 | 4 | 0.773333 | 0.583333 | 143 |
| 4 | 4 | 0.826667 | 0.833333 | 247 |
| 4 | 4 | 0.813333 | 0.916667 | 70 |
| 5 | 5 | 0.52 | 0.166667 | 3 |
| 5 | 5 | 0.66 | 0.5 | 0 |
| 6 | 6 | 0.58 | 0 | 154 |
| 6 | 6 | 0.486667 | 0.166667 | 102 |
| 6 | 6 | 0.346667 | 0.5 | 108 |
| 7 | 7 | 0.78 | 0 | 5 |
| 7 | 7 | 0.62 | 0.083333 | 6 |
| 8 | 8 | 0.713333 | 0 | 55 |
| 8 | 8 | 0.826667 | 0.416667 | 104 |
| 8 | 8 | 0.84 | 0.583333 | 127 |
| 8 | 8 | 0.873333 | 0.75 | 79 |
| 8 | 8 | 0.953333 | 0.833333 | 113 |
| 8 | 8 | 0.913333 | 0.916667 | 60 |
| 8 | 8 | 0.526667 | 1 | 28 |
| 9 | 9 | 0.68 | 0.583333 | 1 |
| 9 | 9 | 0.446667 | 0.75 | 0 |
| 9 | 9 | 0.7 | 0.833333 | 1 |
| 9 | 9 | 0.713333 | 0.916667 | 3 |
| 9 | 9 | 0.64 | 1 | 3 |
| 10 | 10 | 0.033333 | 0.583333 | 8 |
| 10 | 10 | 0.92 | 0.75 | 0 |
| 10 | 10 | 0.64 | 1 | 0 |
| 11 | 11 | 0.493333 | 0 | 49 |
| 11 | 11 | 0.546667 | 0.083333 | 43 |
| 11 | 11 | 0.56 | 0.166667 | 43 |
| 11 | 11 | 0.646667 | 0.25 | 32 |
| 11 | 11 | 0.46 | 0.333333 | 34 |
| 11 | 11 | 0.64 | 0.416667 | 26 |
| 11 | 11 | 0.533333 | 0.5 | 39 |
| 11 | 11 | 0.473333 | 0.666667 | 37 |
| 11 | 11 | 0.56 | 0.833333 | 17 |
| 12 | 12 | 0.6 | 0 | 25 |
| 12 | 12 | 0.586667 | 0.333333 | 9 |
| 12 | 12 | 0.453333 | 0.916667 | 6 |
| 12 | 12 | 0.586667 | 1 | 1 |
| 13 | 13 | 0.76 | 0.583333 | 39 |
| 13 | 13 | 0.946667 | 0.75 | 1 |
| 14 | 14 | 0.533333 | 0 | 79 |
| 14 | 14 | 0.346667 | 0.166667 | 57 |
| 14 | 14 | 0.473333 | 0.333333 | 58 |
| 14 | 14 | 0.3 | 0.5 | 82 |
| 14 | 14 | 0.52 | 0.666667 | 84 |
| 14 | 14 | 0.346667 | 0.833333 | 31 |
| 14 | 14 | 0.6 | 0.916667 | 7 |
| 14 | 14 | 0.993333 | 1 | 35 |
| 15 | 15 | 0.8 | 0.583333 | 16 |
| 15 | 15 | 0.58 | 0.916667 | 6 |
| 16 | 16 | 0.553333 | 0.083333 | 92 |
| 16 | 16 | 0.58 | 0.666667 | 55 |
| 17 | 17 | 0.546667 | 0.083333 | 122 |
| 17 | 17 | 0.56 | 0.166667 | 137 |
| 17 | 17 | 0.46 | 0.333333 | 125 |
| 17 | 17 | 0.426667 | 0.416667 | 142 |
| 17 | 17 | 0.533333 | 0.5 | 186 |
| 17 | 17 | 0.473333 | 0.666667 | 208 |
| 17 | 17 | 0.98 | 0.75 | 159 |
| 17 | 17 | 0.56 | 0.833333 | 199 |
| 17 | 17 | 0.826667 | 0.916667 | 181 |
| 17 | 17 | 0.473333 | 1 | 200 |
| 18 | 18 | 0.933333 | 0.75 | 7 |
| 18 | 18 | 0.826667 | 0.916667 | 3 |
| 18 | 18 | 0.96 | 1 | 2 |
| 19 | 19 | 0.62 | 0.666667 | 65 |
| 19 | 19 | 0.786667 | 0.916667 | 79 |
| 19 | 19 | 0.826667 | 1 | 48 |
| 20 | 20 | 0.686667 | 0 | 16 |
| 20 | 20 | 0.453333 | 0.083333 | 6 |
| 20 | 20 | 0.493333 | 0.166667 | 26 |
| 20 | 20 | 0.766667 | 0.25 | 132 |
| 20 | 20 | 0.413333 | 0.583333 | 16 |
| 20 | 20 | 0.773333 | 0.833333 | 18 |
| 20 | 20 | 0.86 | 0.916667 | 4 |
| 21 | 21 | 0.62 | 0.666667 | 145 |
| 21 | 21 | 0.786667 | 0.916667 | 166 |
| 21 | 21 | 0.913333 | 1 | 229 |
| 22 | 22 | 0.633333 | 0 | 4 |
| 22 | 22 | 0.746667 | 0.166667 | 22 |
| 22 | 22 | 0.753333 | 0.333333 | 3 |
| 22 | 22 | 0.76 | 0.5 | 6 |
| 22 | 22 | 0.746667 | 0.666667 | 4 |
| 23 | 23 | 0.58 | 0.083333 | 28 |
| 23 | 23 | 0.84 | 0.25 | 0 |
| 24 | 24 | 0.68 | 0.083333 | 0 |
| 24 | 24 | 0.586667 | 0.5 | 3 |
| 24 | 24 | 0.606667 | 0.583333 | 10 |
| 24 | 24 | 0.693333 | 0.666667 | 14 |
| 24 | 24 | 0.426667 | 0.75 | 0 |
| 25 | 25 | 0.62 | 0.583333 | 6 |
| 25 | 25 | 0.433333 | 0.75 | 17 |
| 25 | 25 | 0.64 | 0.916667 | 26 |
| 26 | 26 | 0.58 | 0 | 36 |
| 26 | 26 | 0.486667 | 0.166667 | 132 |
| 26 | 26 | 0.866667 | 0.416667 | 3 |
| 26 | 26 | 0.346667 | 0.5 | 27 |
| 26 | 26 | 0.3 | 0.666667 | 48 |
| 26 | 26 | 0.466667 | 0.833333 | 31 |
| 27 | 27 | 0.86 | 0.083333 | 91 |
| 27 | 27 | 0.606667 | 0.333333 | 140 |
| 27 | 27 | 0.673333 | 0.5 | 193 |
| 27 | 27 | 0.806667 | 0.666667 | 151 |
| 27 | 27 | 0.64 | 0.833333 | 133 |
| 27 | 27 | 0.713333 | 1 | 8 |
| 28 | 28 | 0.586667 | 0 | 25 |
| 28 | 28 | 0.7 | 0.166667 | 99 |
| 28 | 28 | 0.553333 | 0.333333 | 39 |
| 28 | 28 | 0.713333 | 0.5 | 46 |
| 28 | 28 | 0.746667 | 0.666667 | 67 |
| 28 | 28 | 0.826667 | 0.833333 | 53 |
| 29 | 29 | 0.726667 | 0.083333 | 55 |
| 29 | 29 | 0.66 | 0.583333 | 33 |
| 29 | 29 | 0.553333 | 0.833333 | 62 |
| 29 | 29 | 0.92 | 1 | 1 |
| 30 | 30 | 0.633333 | 0.666667 | 95 |
| 30 | 30 | 0.726667 | 1 | 31 |
| 31 | 31 | 0.78 | 0.083333 | 4 |
| 31 | 31 | 0.653333 | 0.25 | 25 |
| 31 | 31 | 0.926667 | 0.5 | 36 |
| 31 | 31 | 0.906667 | 0.916667 | 7 |
| 32 | 32 | 0.713333 | 0.083333 | 2 |
| 32 | 32 | 0.666667 | 0.25 | 37 |
| 32 | 32 | 0.726667 | 0.416667 | 64 |
| 32 | 32 | 0.7 | 0.75 | 6 |
| 33 | 33 | 0.653333 | 0.75 | 137 |
| 33 | 33 | 0.626667 | 0.833333 | 31 |
| 33 | 33 | -0.01333 | 0.916667 | 100 |
| 34 | 34 | 0.533333 | 0.083333 | 219 |
| 34 | 34 | 0.566667 | 0.25 | 308 |
| 34 | 34 | 0.513333 | 0.416667 | 211 |
| 34 | 34 | 0.493333 | 0.583333 | 260 |
| 34 | 34 | 0.6 | 0.75 | 162 |
| 35 | 35 | 0.9 | 0.916667 | 0 |
| 35 | 35 | 0.773333 | 1 | 75 |
| 36 | 36 | 0.693333 | 0.166667 | 6 |
| 36 | 36 | 0.606667 | 0.583333 | 0 |
| 37 | 37 | 0.693333 | 0.166667 | 0 |
| 37 | 37 | 0.606667 | 0.583333 | 2 |
| 38 | 38 | 0.746667 | 0.25 | 76 |
| 38 | 38 | 0.78 | 0.416667 | 49 |
| 38 | 38 | 0.8 | 0.583333 | 167 |
| 38 | 38 | 0.646667 | 0.75 | 139 |
| 38 | 38 | 0.86 | 0.833333 | 40 |
| 38 | 38 | -0.02 | 0.916667 | 108 |
| 38 | 38 | 0.906667 | 1 | 72 |
| 39 | 39 | 0.66 | 0 | 89 |
| 39 | 39 | 0.78 | 0.166667 | 37 |
| 39 | 39 | 0.693333 | 0.833333 | 61 |
| 40 | 40 | 0.593333 | 0.583333 | 350 |
| 40 | 40 | 0.906667 | 1 | 254 |
| 41 | 41 | 0.48 | 0.166667 | 8 |
| 41 | 41 | 0.72 | 0.916667 | 16 |
| 42 | 42 | 0.68 | 0.083333 | 21 |
| 42 | 42 | 0.786667 | 0.833333 | 15 |
| 42 | 42 | 0.946667 | 0.916667 | 0 |
| 43 | 43 | 0.573333 | 0.166667 | 25 |
| 43 | 43 | 0.58 | 0.416667 | 25 |
| 43 | 43 | 0.493333 | 0.583333 | 19 |
| 44 | 44 | 0.66 | 0.083333 | 591 |
| 44 | 44 | 0.6 | 0.25 | 332 |
| 44 | 44 | 0.493333 | 0.416667 | 386 |
| 44 | 44 | 0.586667 | 0.583333 | 536 |
| 44 | 44 | 0.566667 | 0.75 | 488 |
| 45 | 45 | 0.566667 | 0.416667 | 46 |
| 45 | 45 | 0.926667 | 0.833333 | 9 |
| 46 | 46 | 0.806667 | 0.75 | 2 |
| 46 | 46 | 0.72 | 0.833333 | 0 |
| 46 | 46 | 0.913333 | 0.916667 | 0 |
| 46 | 46 | 0.586667 | 1 | 0 |
| 47 | 47 | 0.54 | 0.083333 | 11 |
| 47 | 47 | 0.84 | 0.25 | 7 |
| 47 | 47 | 0.606667 | 0.416667 | 6 |
| 47 | 47 | 0.533333 | 0.583333 | 0 |
| 48 | 48 | 0.606667 | 0.333333 | 103 |
| 48 | 48 | 0.666667 | 0.5 | 21 |
| 48 | 48 | 0.693333 | 0.75 | 0 |
| 49 | 49 | 0.72 | 0.083333 | 11 |
| 49 | 49 | 0.753333 | 0.25 | 9 |
| 49 | 49 | 0.726667 | 0.416667 | 13 |
| 49 | 49 | 0.326667 | 0.583333 | 11 |
| 49 | 49 | 0.66 | 0.75 | 9 |
| 50 | 50 | 0.76 | 0 | 1 |
| 50 | 50 | 0.753333 | 0.166667 | 0 |
| 50 | 50 | 0.78 | 0.333333 | 1 |
| 50 | 50 | 0.76 | 0.5 | 0 |
| 50 | 50 | 0.793333 | 0.666667 | 0 |
| 50 | 50 | 0.766667 | 0.833333 | 0 |
| 50 | 50 | 0.726667 | 0.916667 | 2 |
| 50 | 50 | 0.8 | 1 | 0 |
| 51 | 51 | 0.76 | 0 | 2 |
| 51 | 51 | 0.753333 | 0.166667 | 0 |
| 51 | 51 | 0.78 | 0.333333 | 0 |
| 52 | 52 | 0.693333 | 0.916667 | 200 |
| 52 | 52 | 0.72 | 1 | 300 |
| 53 | 53 | 0.72 | 0.083333 | 1093 |
| 53 | 53 | 0.746667 | 0.25 | 1073 |
| 53 | 53 | 0.733333 | 0.416667 | 1042 |
| 53 | 53 | 0.713333 | 0.583333 | 1286 |
| 53 | 53 | 0.693333 | 0.75 | 0 |
| 53 | 53 | 0.773333 | 0.916667 | 1258 |
| 54 | 54 | 0.853333 | 0.916667 | 56 |
| 54 | 54 | 0.773333 | 1 | 81 |
| 55 | 55 | 0.613333 | 0.166667 | 4 |
| 55 | 55 | 0.533333 | 0.583333 | 2 |
| 56 | 56 | 0.666667 | 0 | 39 |
| 56 | 56 | 0.9 | 0.166667 | 29 |
| 56 | 56 | 0.793333 | 0.333333 | 29 |
| 56 | 56 | 0.82 | 0.5 | 45 |
| 56 | 56 | 0.893333 | 0.666667 | 9 |
| 56 | 56 | 0.8 | 0.75 | 7 |
| 56 | 56 | 0.773333 | 0.833333 | 12 |
| 56 | 56 | 0.826667 | 0.916667 | 9 |
| 56 | 56 | 0.733333 | 1 | 9 |
| 57 | 57 | 0.753333 | 0.083333 | 0 |
| 57 | 57 | 0.553333 | 0.333333 | 24 |
| 57 | 57 | 0.953333 | 1 | 15 |
| 58 | 58 | 0.54 | 0.666667 | 101 |
| 58 | 58 | 0.673333 | 0.75 | 3 |
| 59 | 59 | 0.773333 | 0.083333 | 0 |
| 59 | 59 | 0.673333 | 0.916667 | 4 |
| 60 | 60 | 0.773333 | 0.083333 | 16 |
| 60 | 60 | 0.673333 | 0.916667 | 3 |
| 61 | 61 | 0.773333 | 0.166667 | 138 |
| 61 | 61 | 0.82 | 0.5 | 186 |
| 61 | 61 | 0.773333 | 0.666667 | 146 |
| 61 | 61 | 0.68 | 0.916667 | 35 |
| 61 | 62 | 0.793333 | 0 | 27 |
| 61 | 62 | 0.773333 | 0.166667 | 19 |
| 61 | 63 | 0.793333 | 0 | 0 |
| 61 | 63 | 0.773333 | 0.166667 | 17 |
| 61 | 63 | 0.82 | 0.5 | 27 |
| 61 | 63 | 0.773333 | 0.666667 | 14 |
| 61 | 63 | 0.68 | 0.916667 | 0 |
| 61 | 64 | 0.793333 | 0 | 27 |
| 61 | 64 | 0.773333 | 0.166667 | 1 |
| 61 | 64 | 0.82 | 0.5 | 37 |
| 61 | 64 | 0.773333 | 0.666667 | 66 |
| 61 | 64 | 0.68 | 0.916667 | 16 |
| 61 | 65 | 0.773333 | 0.666667 | 2 |
| 61 | 65 | 0.68 | 0.916667 | 2 |
| 61 | 66 | 0.82 | 0.5 | 37 |
| 61 | 66 | 0.773333 | 0.666667 | 56 |
| 62 | 67 | 0.606667 | 0.166667 | 36 |
| 62 | 67 | 0.806667 | 0.333333 | 17 |
| 63 | 68 | 0.433333 | 0 | 412 |
| 63 | 68 | 0.66 | 0.166667 | 414 |
| 63 | 68 | 0.66 | 0.333333 | 225 |
| 63 | 68 | 0.76 | 0.5 | 336 |
| 63 | 68 | 0.626667 | 0.666667 | 485 |
| 63 | 68 | 0.6 | 0.833333 | 186 |
| 63 | 68 | 0.493333 | 1 | 140 |
| 64 | 69 | 0.92 | 0 | 37 |
| 64 | 69 | 0.726667 | 0.083333 | 79 |
| 64 | 69 | 0.733333 | 0.416667 | 1 |
| 64 | 69 | 0.706667 | 0.583333 | 55 |
| 64 | 69 | 0.74 | 0.75 | 19 |
| 64 | 69 | 0.773333 | 0.833333 | 1 |
| 65 | 70 | 0.693333 | 0.833333 | 8 |
| 65 | 70 | 0.673333 | 0.916667 | 19 |
| 66 | 71 | 0.88 | 0.416667 | 38 |
| 66 | 71 | 0.873333 | 0.5 | 42 |
| 66 | 71 | 0.746667 | 0.583333 | 86 |
| 66 | 71 | 0.74 | 0.666667 | 62 |
| 66 | 71 | 0.446667 | 0.75 | 6 |
| 66 | 71 | 0.7 | 0.833333 | 4 |
| 66 | 71 | 0.646667 | 0.916667 | 6 |
| 66 | 71 | 0.64 | 1 | 4 |
| 67 | 72 | 0.44 | 0.083333 | 42 |
| 67 | 72 | 0.52 | 0.25 | 51 |
| 67 | 72 | 0.553333 | 0.416667 | 48 |
| 67 | 72 | 0.533333 | 0.583333 | 30 |
| 67 | 72 | 0.573333 | 0.75 | 25 |
| 68 | 73 | 0.62 | 0 | 1 |
| 68 | 73 | 0.626667 | 0.166667 | 0 |
| 68 | 73 | 0.78 | 0.333333 | 0 |
| 68 | 73 | 0.813333 | 0.916667 | 2 |
| 68 | 73 | 0.713333 | 1 | 2 |
| 69 | 74 | 0.733333 | 0 | 3 |
| 69 | 74 | 0.74 | 1 | 4 |
| 70 | 75 | 0.733333 | 0 | 8 |
| 70 | 75 | 0.74 | 1 | 16 |
| 71 | 76 | 0.833333 | 0.25 | 6 |
| 71 | 76 | 0.633333 | 0.5 | 0 |
| 72 | 77 | 0.833333 | 0.25 | 3 |
| 72 | 77 | 0.633333 | 0.5 | 9 |
| 73 | 78 | 0.533333 | 0.083333 | 8 |
| 73 | 78 | 0.566667 | 0.25 | 1 |
| 73 | 78 | 0.513333 | 0.416667 | 3 |
| 73 | 78 | 0.493333 | 0.583333 | 1 |
| 74 | 79 | 0.533333 | 0.083333 | 13 |
| 74 | 79 | 0.566667 | 0.25 | 5 |
| 74 | 79 | 0.513333 | 0.416667 | 2 |
| 74 | 79 | 0.493333 | 0.583333 | 2 |
| 74 | 79 | 0.6 | 0.75 | 1 |
| 75 | 80 | 0.68 | 0.666667 | 20 |
| 75 | 80 | 0.94 | 0.75 | 0 |
| 76 | 81 | 0.566667 | 0.833333 | 5 |
| 76 | 81 | 0.806667 | 1 | 0 |
| 77 | 82 | 0.613333 | 0.333333 | 123 |
| 77 | 82 | 0.566667 | 0.666667 | 154 |
| 77 | 82 | 0.78 | 1 | 198 |
| 78 | 83 | 0.266667 | 0.25 | 57 |
| 78 | 83 | 0.88 | 0.75 | 0 |
| 78 | 83 | 0.72 | 0.833333 | 0 |
| 78 | 83 | 0.606667 | 1 | 0 |
| 79 | 84 | 0.88 | 0.25 | 36 |
| 79 | 84 | 0.593333 | 0.5 | 23 |
| 79 | 84 | 0.9 | 0.75 | 82 |
| 80 | 85 | 0.72 | 0 | 3 |
| 80 | 85 | 0.666667 | 0.083333 | 1 |
| 80 | 85 | 0.66 | 0.25 | 0 |
| 80 | 85 | 0.633333 | 0.583333 | 0 |
| 81 | 86 | 0.58 | 0 | 26 |
| 81 | 86 | 0.526667 | 0.166667 | 24 |
| 81 | 86 | 0.613333 | 0.333333 | 8 |
| 81 | 86 | 0.58 | 0.416667 | 33 |
| 81 | 86 | 0.62 | 0.583333 | 13 |
| 81 | 86 | 0.793333 | 0.75 | 35 |
| 81 | 86 | 0.586667 | 0.833333 | 42 |
| 81 | 86 | 0.866667 | 1 | 57 |
| 82 | 87 | 0.593333 | 0 | 30 |
| 82 | 87 | 0.7 | 0.166667 | 27 |
| 82 | 87 | 0.673333 | 0.416667 | 18 |
| 82 | 87 | 0.666667 | 0.583333 | 11 |
| 82 | 87 | 0.56 | 0.833333 | 13 |
| 83 | 88 | 0.826667 | 0.583333 | 13 |
| 83 | 88 | 0.9 | 0.75 | 0 |
| 84 | 89 | 0.906667 | 0.916667 | 1 |
| 84 | 89 | 0.813333 | 1 | 0 |
| 85 | 90 | 0.813333 | 0.916667 | 1 |
| 85 | 90 | 0.726667 | 1 | 3 |
| 86 | 91 | 0.606667 | 0.666667 | 45 |
| 86 | 91 | 1 | 1 | 278 |
| 87 | 92 | 0.693333 | 0.916667 | 200 |
| 87 | 92 | 0.72 | 1 | 350 |
| 88 | 93 | 0.833333 | 0.25 | 10 |
| 88 | 93 | 0.633333 | 0.5 | 1 |
| 89 | 94 | 0.633333 | 0.083333 | 362 |
| 89 | 94 | 0.686667 | 0.25 | 315 |
| 89 | 94 | 0.646667 | 0.416667 | 270 |
| 89 | 94 | 0.666667 | 0.583333 | 397 |
| 89 | 94 | 0.64 | 0.75 | 152 |
| 89 | 94 | 0.673333 | 1 | 162 |
| 90 | 95 | 0.92 | 0.916667 | 12 |
| 90 | 95 | 0.806667 | 1 | 5 |
| 91 | 96 | 0.96 | 0.75 | 32 |
| 91 | 96 | 0.926667 | 0.833333 | 18 |
| 91 | 96 | 0.74 | 0.916667 | 4 |
| 92 | 97 | 0.613333 | 0.333333 | 344 |
| 92 | 97 | 0.68 | 0.5 | 590 |
| 92 | 97 | 0.853333 | 0.666667 | 495 |
| 93 | 98 | 0.433333 | 0 | 234 |
| 93 | 98 | 0.66 | 0.166667 | 94 |
| 93 | 98 | 0.66 | 0.333333 | 123 |
| 93 | 98 | 0.72 | 0.5 | 136 |
| 93 | 98 | 0.606667 | 0.666667 | 102 |
| 93 | 98 | 0.86 | 0.833333 | 56 |
| 93 | 98 | 0.753333 | 1 | 11 |
| 94 | 99 | 0.666667 | 0.916667 | 42 |
| 94 | 99 | 0.773333 | 1 | 258 |
| 95 | 100 | 0.626667 | 0 | 20 |
| 95 | 100 | 0.586667 | 0.083333 | 19 |
| 95 | 100 | 0.82 | 0.25 | 25 |
| 95 | 100 | 0.553333 | 0.333333 | 10 |
| 95 | 100 | 0.626667 | 0.5 | 15 |
| 95 | 100 | 0.906667 | 0.833333 | 22 |
| 95 | 100 | 0.873333 | 1 | 14 |
| 96 | 101 | 0.82 | 0.25 | 1 |
| 96 | 101 | 0.626667 | 0.5 | 0 |
| 97 | 102 | 0.413333 | 0.5 | 146 |
| 97 | 102 | 0.68 | 0.666667 | 55 |
| 97 | 102 | 0.726667 | 0.916667 | 78 |
| 97 | 102 | 0.76 | 1 | 222 |
| 98 | 103 | 0.653333 | 0 | 21 |
| 98 | 103 | 0.606667 | 0.833333 | 23 |
| 99 | 104 | 0.686667 | 0.916667 | 42 |
| 99 | 104 | 0.766667 | 1 | 32 |
| 100 | 105 | 0.633333 | 0.083333 | 36 |
| 100 | 105 | 0.773333 | 0.416667 | 16 |
| 100 | 105 | 0.8 | 0.583333 | 8 |
| 100 | 105 | 0.646667 | 0.75 | 5 |
| 100 | 105 | 0.346667 | 0.833333 | 12 |
| 100 | 105 | 0.9 | 1 | 14 |
| 101 | 106 | 0.713333 | 0.083333 | 430 |
| 101 | 106 | 0.753333 | 0.25 | 332 |
| 101 | 106 | 0.806667 | 0.416667 | 535 |
| 101 | 106 | 0.7 | 0.583333 | 722 |
| 101 | 106 | 0.686667 | 0.75 | 349 |
| 101 | 106 | 0.766667 | 1 | 343 |
| 102 | 107 | 0.726667 | 0.083333 | 83 |
| 102 | 107 | 0.746667 | 0.25 | 63 |
| 102 | 107 | 0.733333 | 0.416667 | 34 |
| 102 | 107 | 0.706667 | 0.583333 | 42 |
| 102 | 107 | 0.686667 | 0.75 | 60 |
| 102 | 107 | 0.853333 | 0.916667 | 43 |
| 102 | 107 | 0.846667 | 1 | 244 |
| 103 | 108 | 0.593333 | 0 | 151 |
| 103 | 108 | 0.533333 | 0.166667 | 154 |
| 103 | 108 | 0.506667 | 0.333333 | 127 |
| 103 | 108 | 0.313333 | 0.5 | 138 |
| 103 | 108 | 0.533333 | 0.666667 | 221 |
| 103 | 108 | 0.686667 | 0.833333 | 159 |
| 104 | 109 | 0.586667 | 0.583333 | 44 |
| 104 | 109 | 0.766667 | 0.666667 | 31 |
| 105 | 110 | 0.713333 | 0.25 | 0 |
| 105 | 110 | 0.753333 | 0.333333 | 1 |
| 105 | 110 | 0.54 | 0.583333 | 1 |
| 106 | 111 | 0.713333 | 0.083333 | 535 |
| 106 | 111 | 0.74 | 0.25 | 640 |
| 106 | 111 | 0.726667 | 0.416667 | 578 |
| 106 | 111 | 0.706667 | 0.583333 | 827 |
| 106 | 111 | 0.673333 | 0.75 | 570 |
| 107 | 112 | 0.533333 | 0 | 1 |
| 107 | 112 | 0.833333 | 0.25 | 1 |
| 107 | 112 | 0.693333 | 0.333333 | 1 |
| 107 | 112 | 0.64 | 0.5 | 2 |
| 108 | 113 | 0.813333 | 0.916667 | 58 |
| 108 | 113 | 0.726667 | 1 | 54 |
| 109 | 114 | 0.62 | 0.666667 | 195 |
| 109 | 114 | 0.826667 | 0.916667 | 167 |
| 110 | 115 | 0.573333 | 0.083333 | 161 |
| 110 | 115 | 0.76 | 0.25 | 131 |
| 110 | 115 | 0.706667 | 0.5 | 79 |
| 110 | 115 | 0.753333 | 0.666667 | 62 |
| 110 | 115 | 0.866667 | 0.833333 | 49 |
| 110 | 115 | 0.94 | 1 | 40 |
| 111 | 116 | 0.726667 | 0 | 672 |
| 111 | 116 | 0.66 | 0.083333 | 672 |
| 111 | 116 | 0.653333 | 0.25 | 655 |
| 111 | 116 | 0.56 | 0.416667 | 575 |
| 111 | 116 | 0.673333 | 0.583333 | 555 |
| 111 | 116 | 0.513333 | 0.666667 | 656 |
| 111 | 116 | 0.5 | 0.75 | 722 |
| 111 | 116 | 1 | 0.916667 | 810 |
| 112 | 117 | 0.573333 | 0 | 166 |
| 112 | 117 | 0.66 | 0.166667 | 117 |
| 112 | 117 | 0.746667 | 0.333333 | 141 |
| 112 | 117 | 0.826667 | 0.5 | 31 |
| 113 | 118 | 0.793333 | 0 | 55 |
| 113 | 118 | 0.566667 | 0.75 | 2 |
| 113 | 118 | 0.84 | 0.833333 | 2 |
| 113 | 118 | 0.88 | 0.916667 | 0 |
| 114 | 119 | 0.606667 | 0 | 20 |
| 114 | 119 | 0.673333 | 0.166667 | 20 |
| 114 | 119 | 0.7 | 0.333333 | 47 |
| 114 | 119 | 0.626667 | 0.5 | 16 |
| 114 | 119 | 0.713333 | 0.666667 | 28 |
| 114 | 119 | 0.733333 | 0.833333 | 10 |
| 114 | 119 | 0.813333 | 0.916667 | 32 |
| 114 | 119 | 0.826667 | 1 | 28 |
| 115 | 120 | 0.62 | 0 | 65 |
| 115 | 120 | 0.74 | 0.166667 | 137 |
| 115 | 120 | 0.546667 | 0.333333 | 88 |
| 115 | 120 | 0.813333 | 0.5 | 108 |
| 115 | 120 | 0.673333 | 0.666667 | 122 |
| 115 | 120 | 0.753333 | 0.833333 | 62 |
| 116 | 121 | 0.673333 | 0 | 69 |
| 116 | 121 | 0.613333 | 0.25 | 124 |
| 116 | 121 | 0.6 | 0.416667 | 101 |
| 116 | 121 | 0.766667 | 0.583333 | 99 |
| 116 | 121 | 0.706667 | 0.75 | 12 |
| 117 | 122 | 0.626667 | 0 | 2 |
| 117 | 122 | 0.586667 | 0.083333 | 10 |
| 117 | 122 | 0.626667 | 0.5 | 39 |
| 117 | 122 | 0.76 | 0.666667 | 39 |
| 117 | 122 | 0.74 | 0.833333 | 62 |
| 117 | 122 | 0.96 | 0.916667 | 31 |
| 118 | 123 | 0.666667 | 0.166667 | 0 |
| 118 | 123 | 0.953333 | 0.333333 | 3 |
| 118 | 123 | 0.893333 | 0.75 | 8 |
| 118 | 123 | 0.986667 | 1 | 0 |
| 119 | 124 | 0.426667 | 0 | 135 |
| 119 | 124 | 0.666667 | 0.166667 | 192 |
| 119 | 124 | 0.546667 | 0.333333 | 193 |
| 119 | 124 | 0.76 | 0.5 | 371 |
| 119 | 124 | 0.606667 | 0.666667 | 54 |
| 119 | 124 | 0.766667 | 0.833333 | 41 |
| 119 | 124 | 0.82 | 0.916667 | 57 |
| 120 | 125 | 0.633333 | 0.083333 | 1693 |
| 120 | 125 | 0.686667 | 0.25 | 2145 |
| 120 | 125 | 0.646667 | 0.416667 | 1863 |
| 120 | 125 | 0.666667 | 0.583333 | 2362 |
| 120 | 125 | 0.64 | 0.75 | 1418 |
| 120 | 125 | 0.733333 | 0.916667 | 2024 |
| 120 | 125 | 0.813333 | 1 | 922 |
| 121 | 126 | 0.653333 | 0.333333 | 16 |
| 121 | 126 | 0.673333 | 0.5 | 36 |
| 121 | 126 | 0.66 | 0.666667 | 48 |
| 121 | 126 | 0.646667 | 0.833333 | 16 |
| 121 | 126 | 0.34 | 1 | 1 |
| 122 | 127 | 0.673333 | 0.5 | 7 |
| 122 | 127 | 0.646667 | 0.833333 | 19 |
| 122 | 127 | 0.493333 | 0.916667 | 3 |
| 123 | 128 | 0.846667 | 0.5 | 26 |
| 123 | 128 | 0.686667 | 0.75 | 5 |
| 123 | 128 | 0.813333 | 0.833333 | 7 |
| 123 | 128 | 0.853333 | 0.916667 | 5 |
| 123 | 128 | 0.706667 | 1 | 6 |
| 124 | 129 | 0.546667 | 0 | 22 |
| 124 | 129 | 0.52 | 0.166667 | 10 |
| 124 | 129 | 0.733333 | 0.416667 | 13 |
| 124 | 129 | 0.653333 | 0.583333 | 5 |
| 125 | 130 | 0.506667 | 0 | 325 |
| 125 | 130 | 0.626667 | 0.166667 | 291 |
| 125 | 130 | 0.686667 | 0.416667 | 253 |
| 125 | 130 | 0.633333 | 0.583333 | 327 |
| 125 | 130 | 0.786667 | 0.75 | 258 |
| 126 | 131 | 0.54 | 0.083333 | 1 |
| 126 | 131 | 0.833333 | 0.25 | 1 |
| 127 | 132 | 0.553333 | 0.166667 | 0 |
| 127 | 132 | 0.506667 | 0.833333 | 8 |
| 128 | 133 | 0.666667 | 0.083333 | 3 |
| 128 | 133 | 0.506667 | 0.833333 | 0 |
| 129 | 134 | 0.64 | 0.666667 | 35 |
| 129 | 134 | 0.68 | 1 | 44 |
| 130 | 135 | 0.653333 | 0 | 399 |
| 130 | 135 | 0.68 | 0.166667 | 471 |
| 130 | 135 | 0.666667 | 0.333333 | 313 |
| 130 | 135 | 0.693333 | 0.5 | 541 |
| 130 | 135 | 0.68 | 0.583333 | 0 |
| 130 | 135 | 0.673333 | 0.666667 | 432 |
| 130 | 135 | 0.606667 | 0.833333 | 273 |
| 130 | 135 | 0.82 | 0.916667 | 232 |
| 130 | 135 | 0.8 | 1 | 64 |
| 132 | 137 | 0.5 | 0.083333 | 111 |
| 131 | 136 | 0.62 | 0.25 | 110 |
| 132 | 137 | 0.586667 | 0.416667 | 127 |
| 132 | 137 | 0.486667 | 0.583333 | 131 |
| 132 | 137 | 0.613333 | 0.75 | 87 |
| 133 | 138 | 0.54 | 0 | 3 |
| 133 | 138 | 0.793333 | 0.166667 | 2 |
| 133 | 138 | 0.64 | 0.333333 | 1 |
| 133 | 138 | 0.68 | 0.5 | 0 |
| 133 | 138 | 0.666667 | 0.666667 | 0 |
| 133 | 138 | 0.673333 | 0.833333 | 0 |
| 134 | 139 | 0.633333 | 0.083333 | 250 |
| 134 | 139 | 0.686667 | 0.25 | 272 |
| 134 | 139 | 0.646667 | 0.416667 | 9 |
| 134 | 139 | 0.666667 | 0.583333 | 42 |
| 134 | 139 | 0.64 | 0.75 | 34 |
| 134 | 139 | 0.673333 | 1 | 27 |
| 135 | 140 | 0.953333 | 0 | 13 |
| 135 | 140 | 0.633333 | 0.833333 | 0 |
| 135 | 140 | 0.873333 | 0.916667 | 0 |
| 136 | 141 | 1 | 0.916667 | 240 |
| 136 | 141 | 0.98 | 1 | 148 |
| 137 | 142 | 0.853333 | 0.25 | 34 |
| 137 | 142 | 0.58 | 0.833333 | 24 |
| 137 | 142 | 0.92 | 0.916667 | 0 |
| 138 | 143 | 0.853333 | 0.25 | 0 |
| 138 | 143 | 0.293333 | 0.75 | 1 |
| 138 | 143 | 0.593333 | 0.916667 | 0 |
| 139 | 144 | 0.766667 | 0.666667 | 95 |
| 139 | 144 | 0.92 | 1 | 148 |
| 140 | 145 | 0.666667 | 0 | 76 |
| 140 | 145 | 0.76 | 0.166667 | 78 |
| 140 | 145 | 0.78 | 0.333333 | 66 |
| 140 | 145 | 0.753333 | 0.583333 | 63 |
| 140 | 145 | 0.78 | 0.75 | 39 |
| 140 | 145 | 0.446667 | 0.916667 | 36 |
| 140 | 145 | 0.686667 | 1 | 8 |

***M. sodalis***

| Location | Route | Day | Year | Count |
| --- | --- | --- | --- | --- |
| 1 | 1 | 0.58 | 0 | 2 |
| 1 | 1 | 0.486667 | 0.166667 | 103 |
| 1 | 1 | 0.346667 | 0.5 | 0 |
| 2 | 2 | 0.6 | 0 | 17 |
| 2 | 2 | 0.586667 | 0.333333 | 0 |
| 2 | 2 | 0.453333 | 0.916667 | 0 |
| 2 | 2 | 0.586667 | 1 | 1 |
| 3 | 3 | 0.533333 | 0 | 210 |
| 3 | 3 | 0.346667 | 0.166667 | 240 |
| 3 | 3 | 0.473333 | 0.333333 | 199 |
| 3 | 3 | 0.3 | 0.5 | 243 |
| 3 | 3 | 0.52 | 0.666667 | 262 |
| 3 | 3 | 0.346667 | 0.833333 | 474 |
| 3 | 3 | 0.6 | 0.916667 | 335 |
| 3 | 3 | 0.993333 | 1 | 412 |
| 4 | 4 | 0.546667 | 0.083333 | 0 |
| 4 | 4 | 0.56 | 0.166667 | 0 |
| 4 | 4 | 0.46 | 0.333333 | 0 |
| 4 | 4 | 0.426667 | 0.416667 | 0 |
| 4 | 4 | 0.533333 | 0.5 | 0 |
| 4 | 4 | 0.473333 | 0.666667 | 0 |
| 4 | 4 | 0.98 | 0.75 | 0 |
| 4 | 4 | 0.56 | 0.833333 | 0 |
| 4 | 4 | 0.826667 | 0.916667 | 2 |
| 4 | 4 | 0.473333 | 1 | 3 |
| 5 | 5 | 0.686667 | 0 | 530 |
| 5 | 5 | 0.646667 | 1 | 2491 |
| 6 | 6 | 0.933333 | 0.75 | 30 |
| 6 | 6 | 0.826667 | 0.916667 | 9 |
| 6 | 6 | 0.96 | 1 | 8 |
| 7 | 7 | 0.62 | 0.666667 | 17 |
| 7 | 7 | 0.786667 | 0.916667 | 0 |
| 7 | 7 | 0.826667 | 1 | 19 |
| 8 | 8 | 0.62 | 0.666667 | 61 |
| 8 | 8 | 0.786667 | 0.916667 | 0 |
| 8 | 8 | 0.913333 | 1 | 14 |
| 9 | 9 | 0.633333 | 0 | 158 |
| 9 | 9 | 0.746667 | 0.166667 | 353 |
| 9 | 9 | 0.753333 | 0.333333 | 148 |
| 9 | 9 | 0.76 | 0.5 | 262 |
| 9 | 9 | 0.746667 | 0.666667 | 127 |
| 10 | 10 | 0.58 | 0 | 122 |
| 10 | 10 | 0.486667 | 0.166667 | 98 |
| 10 | 10 | 0.866667 | 0.416667 | 0 |
| 10 | 10 | 0.346667 | 0.5 | 234 |
| 10 | 10 | 0.3 | 0.666667 | 319 |
| 10 | 10 | 0.466667 | 0.833333 | 304 |
| 11 | 11 | 0.86 | 0.083333 | 0 |
| 11 | 11 | 0.606667 | 0.333333 | 1 |
| 11 | 11 | 0.673333 | 0.5 | 0 |
| 11 | 11 | 0.806667 | 0.666667 | 0 |
| 11 | 11 | 0.64 | 0.833333 | 0 |
| 11 | 11 | 0.713333 | 1 | 0 |
| 12 | 12 | 0.586667 | 0 | 0 |
| 12 | 12 | 0.7 | 0.166667 | 0 |
| 12 | 12 | 0.553333 | 0.333333 | 1 |
| 12 | 12 | 0.713333 | 0.5 | 0 |
| 12 | 12 | 0.746667 | 0.666667 | 12 |
| 12 | 12 | 0.826667 | 0.833333 | 43 |
| 13 | 13 | 0.633333 | 0.666667 | 285 |
| 13 | 13 | 0.726667 | 1 | 293 |
| 14 | 14 | 0.78 | 0.083333 | 117 |
| 14 | 14 | 0.653333 | 0.25 | 100 |
| 14 | 14 | 0.926667 | 0.5 | 148 |
| 14 | 14 | 0.906667 | 0.916667 | 183 |
| 15 | 15 | 0.713333 | 0.083333 | 0 |
| 15 | 15 | 0.666667 | 0.25 | 0 |
| 15 | 15 | 0.726667 | 0.416667 | 21 |
| 15 | 15 | 0.7 | 0.75 | 3 |
| 16 | 16 | 0.593333 | 0.583333 | 100 |
| 16 | 16 | 0.906667 | 1 | 235 |
| 17 | 17 | 0.573333 | 0.166667 | 49 |
| 17 | 17 | 0.58 | 0.416667 | 24 |
| 17 | 17 | 0.493333 | 0.583333 | 44 |
| 18 | 18 | 0.66 | 0.083333 | 92 |
| 18 | 18 | 0.6 | 0.25 | 109 |
| 18 | 18 | 0.493333 | 0.416667 | 109 |
| 18 | 18 | 0.586667 | 0.583333 | 139 |
| 18 | 18 | 0.566667 | 0.75 | 188 |
| 19 | 19 | 0.54 | 0.083333 | 0 |
| 19 | 19 | 0.84 | 0.25 | 1 |
| 19 | 19 | 0.606667 | 0.416667 | 0 |
| 19 | 19 | 0.533333 | 0.583333 | 0 |
| 20 | 20 | 0.72 | 0.083333 | 6 |
| 20 | 20 | 0.753333 | 0.25 | 5 |
| 20 | 20 | 0.726667 | 0.416667 | 3 |
| 20 | 20 | 0.326667 | 0.583333 | 2 |
| 20 | 20 | 0.66 | 0.75 | 1 |
| 21 | 21 | 0.76 | 0 | 3129 |
| 21 | 21 | 0.753333 | 0.166667 | 2264 |
| 21 | 21 | 0.78 | 0.333333 | 1704 |
| 21 | 21 | 0.76 | 0.5 | 2065 |
| 21 | 21 | 0.793333 | 0.666667 | 1908 |
| 21 | 21 | 0.766667 | 0.833333 | 1719 |
| 21 | 21 | 0.726667 | 0.916667 | 509 |
| 21 | 21 | 0.8 | 1 | 433 |
| 22 | 22 | 0.72 | 0.083333 | 4 |
| 22 | 22 | 0.746667 | 0.25 | 6 |
| 22 | 22 | 0.733333 | 0.416667 | 3 |
| 22 | 22 | 0.713333 | 0.583333 | 2 |
| 22 | 22 | 0.693333 | 0.75 | 12 |
| 22 | 22 | 0.773333 | 0.916667 | 7 |
| 23 | 23 | 0.666667 | 0 | 345 |
| 23 | 23 | 0.9 | 0.166667 | 500 |
| 23 | 23 | 0.793333 | 0.333333 | 710 |
| 23 | 23 | 0.82 | 0.5 | 685 |
| 23 | 23 | 0.893333 | 0.666667 | 0 |
| 23 | 23 | 0.8 | 0.75 | 0 |
| 23 | 23 | 0.773333 | 0.833333 | 0 |
| 23 | 23 | 0.826667 | 0.916667 | 0 |
| 23 | 23 | 0.733333 | 1 | 0 |
| 24 | 24 | 0.773333 | 0.166667 | 598 |
| 24 | 24 | 0.82 | 0.5 | 201 |
| 24 | 24 | 0.773333 | 0.666667 | 506 |
| 24 | 24 | 0.68 | 0.916667 | 377 |
| 24 | 25 | 0.793333 | 0 | 0 |
| 24 | 25 | 0.773333 | 0.166667 | 0 |
| 24 | 26 | 0.793333 | 0 | 8094 |
| 24 | 26 | 0.773333 | 0.166667 | 7965 |
| 24 | 26 | 0.82 | 0.5 | 11567 |
| 24 | 26 | 0.773333 | 0.666667 | 12151 |
| 24 | 26 | 0.68 | 0.916667 | 17953 |
| 24 | 27 | 0.793333 | 0 | 80 |
| 24 | 27 | 0.773333 | 0.166667 | 3 |
| 24 | 27 | 0.82 | 0.5 | 122 |
| 24 | 27 | 0.773333 | 0.666667 | 200 |
| 24 | 27 | 0.68 | 0.916667 | 227 |
| 24 | 28 | 0.82 | 0.5 | 0 |
| 24 | 28 | 0.773333 | 0.666667 | 1 |
| 25 | 29 | 0.92 | 0 | 40 |
| 25 | 29 | 0.726667 | 0.083333 | 16 |
| 25 | 29 | 0.733333 | 0.416667 | 31 |
| 25 | 29 | 0.706667 | 0.583333 | 41 |
| 25 | 29 | 0.74 | 0.75 | 41 |
| 25 | 29 | 0.773333 | 0.833333 | 42 |
| 26 | 30 | 0.486667 | 0.25 | 0 |
| 26 | 30 | 0.48 | 0.583333 | 0 |
| 26 | 30 | 0.6 | 0.916667 | 51 |
| 26 | 30 | 0.813333 | 1 | 0 |
| 27 | 31 | 0.44 | 0.083333 | 84 |
| 27 | 31 | 0.52 | 0.25 | 94 |
| 27 | 31 | 0.553333 | 0.416667 | 92 |
| 27 | 31 | 0.533333 | 0.583333 | 97 |
| 27 | 31 | 0.573333 | 0.75 | 115 |
| 28 | 32 | 0.62 | 0 | 4015 |
| 28 | 32 | 0.626667 | 0.166667 | 3835 |
| 28 | 32 | 0.78 | 0.333333 | 3371 |
| 28 | 32 | 0.813333 | 0.916667 | 377 |
| 28 | 32 | 0.713333 | 1 | 251 |
| 29 | 33 | 0.58 | 0 | 4 |
| 29 | 33 | 0.526667 | 0.166667 | 1 |
| 29 | 33 | 0.613333 | 0.333333 | 4 |
| 29 | 33 | 0.58 | 0.416667 | 11 |
| 29 | 33 | 0.62 | 0.583333 | 6 |
| 30 | 34 | 0.813333 | 0.916667 | 0 |
| 30 | 34 | 0.726667 | 1 | 5 |
| 31 | 35 | 0.693333 | 0.916667 | 51 |
| 31 | 35 | 0.72 | 1 | 0 |
| 32 | 36 | 0.633333 | 0.083333 | 142 |
| 32 | 36 | 0.686667 | 0.25 | 181 |
| 32 | 36 | 0.646667 | 0.416667 | 196 |
| 32 | 36 | 0.666667 | 0.583333 | 241 |
| 32 | 36 | 0.64 | 0.75 | 251 |
| 32 | 36 | 0.673333 | 1 | 176 |
| 33 | 37 | 0.433333 | 0 | 55 |
| 33 | 37 | 0.66 | 0.166667 | 3 |
| 33 | 37 | 0.66 | 0.333333 | 35 |
| 33 | 37 | 0.72 | 0.5 | 67 |
| 33 | 37 | 0.606667 | 0.666667 | 57 |
| 33 | 37 | 0.86 | 0.833333 | 33 |
| 33 | 37 | 0.753333 | 1 | 10 |
| 34 | 38 | 0.786667 | 0 | 77 |
| 34 | 38 | 0.413333 | 0.5 | 122 |
| 34 | 38 | 0.68 | 0.666667 | 356 |
| 34 | 38 | 0.726667 | 0.916667 | 13 |
| 34 | 38 | 0.76 | 1 | 12 |
| 35 | 39 | 0.713333 | 0.083333 | 3 |
| 35 | 39 | 0.753333 | 0.25 | 11 |
| 35 | 39 | 0.806667 | 0.416667 | 14 |
| 35 | 39 | 0.7 | 0.583333 | 14 |
| 35 | 39 | 0.686667 | 0.75 | 16 |
| 35 | 39 | 0.766667 | 1 | 19 |
| 36 | 40 | 0.726667 | 0.083333 | 8 |
| 36 | 40 | 0.746667 | 0.25 | 10 |
| 36 | 40 | 0.733333 | 0.416667 | 8 |
| 36 | 40 | 0.706667 | 0.583333 | 5 |
| 36 | 40 | 0.686667 | 0.75 | 6 |
| 36 | 40 | 0.853333 | 0.916667 | 8 |
| 36 | 40 | 0.846667 | 1 | 0 |
| 37 | 41 | 0.593333 | 0 | 0 |
| 37 | 41 | 0.533333 | 0.166667 | 0 |
| 37 | 41 | 0.506667 | 0.333333 | 0 |
| 37 | 41 | 0.313333 | 0.5 | 0 |
| 37 | 41 | 0.533333 | 0.666667 | 1 |
| 37 | 41 | 0.686667 | 0.833333 | 0 |
| 38 | 42 | 0.713333 | 0.083333 | 54 |
| 38 | 42 | 0.74 | 0.25 | 40 |
| 38 | 42 | 0.726667 | 0.416667 | 35 |
| 38 | 42 | 0.706667 | 0.583333 | 37 |
| 38 | 42 | 0.673333 | 0.75 | 34 |
| 39 | 43 | 0.813333 | 0.916667 | 2 |
| 39 | 43 | 0.726667 | 1 | 0 |
| 40 | 44 | 1 | 0.333333 | 47 |
| 40 | 44 | 0.62 | 0.666667 | 53 |
| 40 | 44 | 0.826667 | 0.916667 | 32 |
| 41 | 45 | 0.726667 | 0 | 132 |
| 41 | 45 | 0.66 | 0.083333 | 121 |
| 41 | 45 | 0.653333 | 0.25 | 139 |
| 41 | 45 | 0.56 | 0.416667 | 93 |
| 41 | 45 | 0.673333 | 0.583333 | 52 |
| 41 | 45 | 0.513333 | 0.666667 | 44 |
| 41 | 45 | 0.5 | 0.75 | 21 |
| 41 | 45 | 1 | 0.916667 | 23 |
| 42 | 46 | 0.606667 | 0 | 0 |
| 42 | 46 | 0.673333 | 0.166667 | 0 |
| 42 | 46 | 0.7 | 0.333333 | 0 |
| 42 | 46 | 0.626667 | 0.5 | 0 |
| 42 | 46 | 0.713333 | 0.666667 | 1 |
| 42 | 46 | 0.733333 | 0.833333 | 0 |
| 42 | 46 | 0.813333 | 0.916667 | 1 |
| 42 | 46 | 0.826667 | 1 | 0 |
| 43 | 47 | 0.673333 | 0 | 80 |
| 43 | 47 | 0.786667 | 1 | 12 |
| 44 | 48 | 0.62 | 0 | 3 |
| 44 | 48 | 0.74 | 0.166667 | 0 |
| 44 | 48 | 0.546667 | 0.333333 | 1 |
| 44 | 48 | 0.813333 | 0.5 | 0 |
| 44 | 48 | 0.673333 | 0.666667 | 0 |
| 44 | 48 | 0.753333 | 0.833333 | 0 |
| 45 | 49 | 0.633333 | 0.083333 | 140 |
| 45 | 49 | 0.686667 | 0.25 | 140 |
| 45 | 49 | 0.646667 | 0.416667 | 193 |
| 45 | 49 | 0.666667 | 0.583333 | 250 |
| 45 | 49 | 0.64 | 0.75 | 287 |
| 45 | 49 | 0.733333 | 0.916667 | 304 |
| 45 | 49 | 0.813333 | 1 | 179 |
| 46 | 50 | 0.506667 | 0 | 83 |
| 46 | 50 | 0.626667 | 0.166667 | 40 |
| 46 | 50 | 0.686667 | 0.416667 | 36 |
| 46 | 50 | 0.633333 | 0.583333 | 55 |
| 46 | 50 | 0.786667 | 0.75 | 91 |
| 47 | 51 | 0.64 | 0.666667 | 55 |
| 47 | 51 | 0.68 | 1 | 3 |
| 48 | 52 | 0.653333 | 0 | 19 |
| 48 | 52 | 0.68 | 0.166667 | 24 |
| 48 | 52 | 0.666667 | 0.333333 | 25 |
| 48 | 52 | 0.693333 | 0.5 | 95 |
| 48 | 52 | 0.68 | 0.583333 | 93 |
| 48 | 52 | 0.673333 | 0.666667 | 158 |
| 48 | 52 | 0.606667 | 0.833333 | 139 |
| 48 | 52 | 0.82 | 0.916667 | 82 |
| 48 | 52 | 0.8 | 1 | 90 |
| 50 | 54 | 0.5 | 0.083333 | 3 |
| 49 | 53 | 0.62 | 0.25 | 7 |
| 50 | 54 | 0.586667 | 0.416667 | 0 |
| 50 | 54 | 0.486667 | 0.583333 | 1 |
| 50 | 54 | 0.613333 | 0.75 | 10 |
| 51 | 55 | 0.633333 | 0.083333 | 0 |
| 51 | 55 | 0.686667 | 0.25 | 0 |
| 51 | 55 | 0.646667 | 0.416667 | 0 |
| 51 | 55 | 0.666667 | 0.583333 | 0 |
| 51 | 55 | 0.64 | 0.75 | 0 |
| 51 | 55 | 0.673333 | 1 | 4 |
| 52 | 56 | 0.953333 | 0 | 9415 |
| 52 | 56 | 0.633333 | 0.833333 | 341 |
| 52 | 56 | 0.873333 | 0.916667 | 190 |
| 53 | 57 | 0.673333 | 0 | 3000 |
| 53 | 57 | 1 | 0.916667 | 6400 |
| 53 | 57 | 0.98 | 1 | 7495 |
| 54 | 58 | 0.853333 | 0.25 | 0 |
| 54 | 58 | 0.62 | 0.666667 | 0 |
| 54 | 58 | 0.5 | 0.916667 | 0 |
| 54 | 58 | 0.92 | 1 | 1 |
| 55 | 59 | 0.853333 | 0.25 | 0 |
| 55 | 59 | 0.58 | 0.833333 | 3 |
| 55 | 59 | 0.92 | 0.916667 | 1 |
| 56 | 60 | 0.853333 | 0.25 | 0 |
| 56 | 60 | 0.293333 | 0.75 | 0 |
| 56 | 60 | 0.593333 | 0.916667 | 1 |
| 57 | 61 | 0.606667 | 0.166667 | 2415 |
| 57 | 61 | 0.766667 | 0.666667 | 1437 |
| 57 | 61 | 0.92 | 1 | 875 |
| 58 | 62 | 0.68 | 0.666667 | 100 |
| 58 | 62 | 0.953333 | 1 | 53 |

***M. septentrionalis***

| Location | Route | Day | Year | Count |
| --- | --- | --- | --- | --- |
| 1 | 1 | 0.64 | 0 | 23 |
| 1 | 1 | 0.613333 | 0.166667 | 29 |
| 1 | 1 | 0.766667 | 0.416667 | 6 |
| 1 | 1 | 0.793333 | 0.583333 | 8 |
| 1 | 1 | 0.653333 | 0.833333 | 10 |
| 1 | 1 | 0.506667 | 0.916667 | 1 |
| 2 | 2 | 0.613333 | 0.333333 | 11 |
| 2 | 2 | 0.673333 | 0.916667 | 9 |
| 2 | 2 | 0.673333 | 1 | 2 |
| 3 | 3 | 0.613333 | 0.333333 | 0 |
| 3 | 3 | 0.68 | 0.833333 | 12 |
| 4 | 4 | 0.766667 | 0.25 | 17 |
| 4 | 4 | 0.686667 | 0.333333 | 9 |
| 4 | 4 | 0.7 | 0.416667 | 29 |
| 4 | 4 | 0.773333 | 0.583333 | 3 |
| 4 | 4 | 0.826667 | 0.833333 | 15 |
| 4 | 4 | 0.813333 | 0.916667 | 6 |
| 5 | 5 | 0.52 | 0.166667 | 3 |
| 5 | 5 | 0.66 | 0.5 | 0 |
| 6 | 6 | 0.58 | 0 | 1 |
| 6 | 6 | 0.486667 | 0.166667 | 0 |
| 6 | 6 | 0.346667 | 0.5 | 0 |
| 7 | 7 | 0.78 | 0 | 7 |
| 7 | 7 | 0.62 | 0.083333 | 12 |
| 8 | 8 | 0.713333 | 0 | 1 |
| 8 | 8 | 0.826667 | 0.416667 | 4 |
| 8 | 8 | 0.84 | 0.583333 | 1 |
| 8 | 8 | 0.873333 | 0.75 | 1 |
| 8 | 8 | 0.953333 | 0.833333 | 0 |
| 8 | 8 | 0.913333 | 0.916667 | 0 |
| 8 | 8 | 0.526667 | 1 | 0 |
| 9 | 9 | 0.68 | 0.583333 | 6 |
| 9 | 9 | 0.446667 | 0.75 | 0 |
| 9 | 9 | 0.7 | 0.833333 | 0 |
| 9 | 9 | 0.713333 | 0.916667 | 0 |
| 9 | 9 | 0.64 | 1 | 0 |
| 10 | 10 | 0.033333 | 0.583333 | 2 |
| 10 | 10 | 0.92 | 0.75 | 0 |
| 10 | 10 | 0.64 | 1 | 0 |
| 11 | 11 | 0.493333 | 0 | 1 |
| 11 | 11 | 0.546667 | 0.083333 | 2 |
| 11 | 11 | 0.56 | 0.166667 | 0 |
| 11 | 11 | 0.646667 | 0.25 | 0 |
| 11 | 11 | 0.46 | 0.333333 | 0 |
| 11 | 11 | 0.64 | 0.416667 | 0 |
| 11 | 11 | 0.533333 | 0.5 | 0 |
| 11 | 11 | 0.473333 | 0.666667 | 1 |
| 11 | 11 | 0.56 | 0.833333 | 1 |
| 12 | 12 | 0.6 | 0 | 12 |
| 12 | 12 | 0.586667 | 0.333333 | 26 |
| 12 | 12 | 0.453333 | 0.916667 | 11 |
| 12 | 12 | 0.586667 | 1 | 4 |
| 13 | 13 | 0.76 | 0.583333 | 8 |
| 13 | 13 | 0.946667 | 0.75 | 0 |
| 14 | 14 | 0.533333 | 0 | 1 |
| 14 | 14 | 0.346667 | 0.166667 | 4 |
| 14 | 14 | 0.473333 | 0.333333 | 1 |
| 14 | 14 | 0.3 | 0.5 | 1 |
| 14 | 14 | 0.52 | 0.666667 | 2 |
| 14 | 14 | 0.346667 | 0.833333 | 0 |
| 14 | 14 | 0.6 | 0.916667 | 0 |
| 14 | 14 | 0.993333 | 1 | 6 |
| 15 | 15 | 0.553333 | 0.083333 | 12 |
| 15 | 15 | 0.58 | 0.666667 | 3 |
| 16 | 16 | 0.546667 | 0.083333 | 2 |
| 16 | 16 | 0.56 | 0.166667 | 0 |
| 16 | 16 | 0.46 | 0.333333 | 0 |
| 16 | 16 | 0.426667 | 0.416667 | 7 |
| 16 | 16 | 0.533333 | 0.5 | 1 |
| 16 | 16 | 0.473333 | 0.666667 | 2 |
| 16 | 16 | 0.98 | 0.75 | 0 |
| 16 | 16 | 0.56 | 0.833333 | 4 |
| 16 | 16 | 0.826667 | 0.916667 | 4 |
| 16 | 16 | 0.473333 | 1 | 0 |
| 17 | 17 | 0.933333 | 0.75 | 0 |
| 17 | 17 | 0.826667 | 0.916667 | 1 |
| 17 | 17 | 0.96 | 1 | 0 |
| 18 | 18 | 0.62 | 0.666667 | 0 |
| 18 | 18 | 0.786667 | 0.916667 | 0 |
| 18 | 18 | 0.913333 | 1 | 1 |
| 19 | 19 | 0.633333 | 0 | 13 |
| 19 | 19 | 0.746667 | 0.166667 | 32 |
| 19 | 19 | 0.753333 | 0.333333 | 6 |
| 19 | 19 | 0.76 | 0.5 | 8 |
| 19 | 19 | 0.746667 | 0.666667 | 20 |
| 20 | 20 | 0.58 | 0.083333 | 2 |
| 20 | 20 | 0.84 | 0.25 | 0 |
| 21 | 21 | 0.62 | 0.583333 | 0 |
| 21 | 21 | 0.433333 | 0.75 | 2 |
| 21 | 21 | 0.64 | 0.916667 | 2 |
| 22 | 22 | 0.58 | 0 | 0 |
| 22 | 22 | 0.486667 | 0.166667 | 0 |
| 22 | 22 | 0.866667 | 0.416667 | 0 |
| 22 | 22 | 0.346667 | 0.5 | 1 |
| 22 | 22 | 0.3 | 0.666667 | 0 |
| 22 | 22 | 0.466667 | 0.833333 | 0 |
| 23 | 23 | 0.86 | 0.083333 | 0 |
| 23 | 23 | 0.606667 | 0.333333 | 1 |
| 23 | 23 | 0.673333 | 0.5 | 0 |
| 23 | 23 | 0.806667 | 0.666667 | 0 |
| 23 | 23 | 0.64 | 0.833333 | 2 |
| 23 | 23 | 0.713333 | 1 | 0 |
| 24 | 24 | 0.726667 | 0.083333 | 0 |
| 24 | 24 | 0.66 | 0.583333 | 13 |
| 24 | 24 | 0.553333 | 0.833333 | 1 |
| 24 | 24 | 0.92 | 1 | 0 |
| 25 | 25 | 0.633333 | 0.666667 | 0 |
| 25 | 25 | 0.726667 | 1 | 1 |
| 26 | 26 | 0.713333 | 0.083333 | 0 |
| 26 | 26 | 0.666667 | 0.25 | 0 |
| 26 | 26 | 0.726667 | 0.416667 | 30 |
| 26 | 26 | 0.7 | 0.75 | 8 |
| 27 | 27 | 0.653333 | 0.75 | 26 |
| 27 | 27 | 0.626667 | 0.833333 | 1 |
| 27 | 27 | -0.01333 | 0.916667 | 0 |
| 28 | 28 | 0.533333 | 0.083333 | 2 |
| 28 | 28 | 0.566667 | 0.25 | 2 |
| 28 | 28 | 0.513333 | 0.416667 | 4 |
| 28 | 28 | 0.493333 | 0.583333 | 1 |
| 28 | 28 | 0.6 | 0.75 | 1 |
| 29 | 29 | 0.9 | 0.916667 | 1 |
| 29 | 29 | 0.773333 | 1 | 5 |
| 30 | 30 | 0.746667 | 0.25 | 556 |
| 30 | 30 | 0.78 | 0.416667 | 265 |
| 30 | 30 | 0.8 | 0.583333 | 881 |
| 30 | 30 | 0.646667 | 0.75 | 553 |
| 30 | 30 | 0.86 | 0.833333 | 44 |
| 30 | 30 | -0.02 | 0.916667 | 254 |
| 30 | 30 | 0.906667 | 1 | 306 |
| 31 | 31 | 0.66 | 0 | 0 |
| 31 | 31 | 0.78 | 0.166667 | 2 |
| 31 | 31 | 0.693333 | 0.833333 | 1 |
| 32 | 32 | 0.48 | 0.166667 | 27 |
| 32 | 32 | 0.72 | 0.916667 | 17 |
| 33 | 33 | 0.68 | 0.083333 | 15 |
| 33 | 33 | 0.786667 | 0.833333 | 2 |
| 33 | 33 | 0.946667 | 0.916667 | 0 |
| 34 | 34 | 0.573333 | 0.166667 | 1 |
| 34 | 34 | 0.58 | 0.416667 | 0 |
| 34 | 34 | 0.493333 | 0.583333 | 0 |
| 35 | 35 | 0.66 | 0.083333 | 6 |
| 35 | 35 | 0.6 | 0.25 | 2 |
| 35 | 35 | 0.493333 | 0.416667 | 1 |
| 35 | 35 | 0.586667 | 0.583333 | 1 |
| 35 | 35 | 0.566667 | 0.75 | 1 |
| 36 | 36 | 0.566667 | 0.416667 | 26 |
| 36 | 36 | 0.926667 | 0.833333 | 0 |
| 37 | 37 | 0.54 | 0.083333 | 1 |
| 37 | 37 | 0.84 | 0.25 | 0 |
| 37 | 37 | 0.606667 | 0.416667 | 0 |
| 37 | 37 | 0.533333 | 0.583333 | 0 |
| 38 | 38 | 0.72 | 0.083333 | 0 |
| 38 | 38 | 0.753333 | 0.25 | 0 |
| 38 | 38 | 0.726667 | 0.416667 | 1 |
| 38 | 38 | 0.326667 | 0.583333 | 0 |
| 38 | 38 | 0.66 | 0.75 | 0 |
| 39 | 39 | 0.693333 | 0.916667 | 0 |
| 39 | 39 | 0.72 | 1 | 2 |
| 40 | 40 | 0.72 | 0.083333 | 3 |
| 40 | 40 | 0.746667 | 0.25 | 2 |
| 40 | 40 | 0.733333 | 0.416667 | 3 |
| 40 | 40 | 0.713333 | 0.583333 | 1 |
| 40 | 40 | 0.693333 | 0.75 | 1 |
| 40 | 40 | 0.773333 | 0.916667 | 0 |
| 41 | 41 | 0.853333 | 0.916667 | 136 |
| 41 | 41 | 0.773333 | 1 | 0 |
| 42 | 42 | 0.666667 | 0 | 5 |
| 42 | 42 | 0.9 | 0.166667 | 9 |
| 42 | 42 | 0.793333 | 0.333333 | 39 |
| 42 | 42 | 0.82 | 0.5 | 14 |
| 42 | 42 | 0.893333 | 0.666667 | 5 |
| 42 | 42 | 0.8 | 0.75 | 0 |
| 42 | 42 | 0.773333 | 0.833333 | 1 |
| 42 | 42 | 0.826667 | 0.916667 | 0 |
| 42 | 42 | 0.733333 | 1 | 4 |
| 43 | 43 | 0.753333 | 0.083333 | 0 |
| 43 | 43 | 0.553333 | 0.333333 | 2 |
| 43 | 43 | 0.953333 | 1 | 1 |
| 44 | 44 | 0.54 | 0.666667 | 12 |
| 44 | 44 | 0.673333 | 0.75 | 1 |
| 45 | 45 | 0.793333 | 0 | 0 |
| 45 | 45 | 0.773333 | 0.166667 | 1 |
| 45 | 45 | 0.82 | 0.5 | 0 |
| 45 | 45 | 0.773333 | 0.666667 | 0 |
| 45 | 45 | 0.68 | 0.916667 | 0 |
| 46 | 46 | 0.606667 | 0.166667 | 3 |
| 46 | 46 | 0.806667 | 0.333333 | 0 |
| 47 | 47 | 0.433333 | 0 | 0 |
| 47 | 47 | 0.66 | 0.166667 | 0 |
| 47 | 47 | 0.66 | 0.333333 | 0 |
| 47 | 47 | 0.76 | 0.5 | 1 |
| 47 | 47 | 0.626667 | 0.666667 | 4 |
| 47 | 47 | 0.6 | 0.833333 | 0 |
| 47 | 47 | 0.493333 | 1 | 3 |
| 48 | 48 | 0.92 | 0 | 0 |
| 48 | 48 | 0.726667 | 0.083333 | 0 |
| 48 | 48 | 0.733333 | 0.416667 | 1 |
| 48 | 48 | 0.706667 | 0.583333 | 0 |
| 48 | 48 | 0.74 | 0.75 | 0 |
| 48 | 48 | 0.773333 | 0.833333 | 0 |
| 49 | 49 | 0.693333 | 0.833333 | 45 |
| 49 | 49 | 0.673333 | 0.916667 | 1 |
| 50 | 50 | 0.88 | 0.416667 | 5 |
| 50 | 50 | 0.873333 | 0.5 | 5 |
| 50 | 50 | 0.746667 | 0.583333 | 10 |
| 50 | 50 | 0.74 | 0.666667 | 0 |
| 50 | 50 | 0.446667 | 0.75 | 1 |
| 50 | 50 | 0.7 | 0.833333 | 0 |
| 50 | 50 | 0.646667 | 0.916667 | 0 |
| 50 | 50 | 0.64 | 1 | 0 |
| 51 | 51 | 0.44 | 0.083333 | 0 |
| 51 | 51 | 0.52 | 0.25 | 0 |
| 51 | 51 | 0.553333 | 0.416667 | 2 |
| 51 | 51 | 0.533333 | 0.583333 | 0 |
| 51 | 51 | 0.573333 | 0.75 | 0 |
| 52 | 52 | 0.62 | 0 | 0 |
| 52 | 52 | 0.626667 | 0.166667 | 0 |
| 52 | 52 | 0.78 | 0.333333 | 2 |
| 52 | 52 | 0.813333 | 0.916667 | 1 |
| 52 | 52 | 0.713333 | 1 | 1 |
| 53 | 53 | 0.733333 | 0 | 24 |
| 53 | 53 | 0.74 | 1 | 5 |
| 54 | 54 | 0.733333 | 0 | 1 |
| 54 | 54 | 0.74 | 1 | 6 |
| 55 | 55 | 0.833333 | 0.25 | 0 |
| 55 | 55 | 0.633333 | 0.5 | 0 |
| 56 | 56 | 0.833333 | 0.25 | 1 |
| 56 | 56 | 0.633333 | 0.5 | 0 |
| 57 | 57 | 0.533333 | 0.083333 | 0 |
| 57 | 57 | 0.566667 | 0.25 | 0 |
| 57 | 57 | 0.513333 | 0.416667 | 0 |
| 57 | 57 | 0.493333 | 0.583333 | 0 |
| 57 | 57 | 0.6 | 0.75 | 1 |
| 58 | 58 | 0.566667 | 0.833333 | 1 |
| 58 | 58 | 0.806667 | 1 | 0 |
| 59 | 59 | 0.613333 | 0.333333 | 2 |
| 59 | 59 | 0.566667 | 0.666667 | 0 |
| 59 | 59 | 0.78 | 1 | 1 |
| 60 | 60 | 0.266667 | 0.25 | 4 |
| 60 | 60 | 0.88 | 0.75 | 0 |
| 60 | 60 | 0.72 | 0.833333 | 0 |
| 60 | 60 | 0.606667 | 1 | 0 |
| 61 | 61 | 0.88 | 0.25 | 6 |
| 61 | 61 | 0.593333 | 0.5 | 9 |
| 61 | 61 | 0.9 | 0.75 | 5 |
| 62 | 62 | 0.58 | 0 | 0 |
| 62 | 62 | 0.526667 | 0.166667 | 0 |
| 62 | 62 | 0.613333 | 0.333333 | 0 |
| 62 | 62 | 0.58 | 0.416667 | 0 |
| 62 | 62 | 0.62 | 0.583333 | 0 |
| 62 | 62 | 0.793333 | 0.75 | 0 |
| 62 | 62 | 0.586667 | 0.833333 | 5 |
| 62 | 62 | 0.866667 | 1 | 30 |
| 63 | 63 | 0.593333 | 0 | 0 |
| 63 | 63 | 0.7 | 0.166667 | 2 |
| 63 | 63 | 0.673333 | 0.416667 | 0 |
| 63 | 63 | 0.666667 | 0.583333 | 2 |
| 63 | 63 | 0.56 | 0.833333 | 1 |
| 64 | 64 | 0.826667 | 0.583333 | 10 |
| 64 | 64 | 0.9 | 0.75 | 0 |
| 65 | 65 | 0.606667 | 0.666667 | 2 |
| 65 | 65 | 1 | 1 | 5 |
| 66 | 66 | 0.833333 | 0.25 | 1 |
| 66 | 66 | 0.633333 | 0.5 | 0 |
| 67 | 67 | 0.633333 | 0.083333 | 1 |
| 67 | 67 | 0.686667 | 0.25 | 1 |
| 67 | 67 | 0.646667 | 0.416667 | 0 |
| 67 | 67 | 0.666667 | 0.583333 | 0 |
| 67 | 67 | 0.64 | 0.75 | 0 |
| 67 | 67 | 0.673333 | 1 | 3 |
| 68 | 68 | 0.96 | 0.75 | 21 |
| 68 | 68 | 0.926667 | 0.833333 | 0 |
| 68 | 68 | 0.74 | 0.916667 | 0 |
| 69 | 69 | 0.613333 | 0.333333 | 0 |
| 69 | 69 | 0.68 | 0.5 | 11 |
| 69 | 69 | 0.853333 | 0.666667 | 2 |
| 70 | 70 | 0.433333 | 0 | 1 |
| 70 | 70 | 0.66 | 0.166667 | 0 |
| 70 | 70 | 0.66 | 0.333333 | 3 |
| 70 | 70 | 0.72 | 0.5 | 1 |
| 70 | 70 | 0.606667 | 0.666667 | 1 |
| 70 | 70 | 0.86 | 0.833333 | 0 |
| 70 | 70 | 0.753333 | 1 | 0 |
| 71 | 71 | 0.666667 | 0.916667 | 3 |
| 71 | 71 | 0.773333 | 1 | 0 |
| 72 | 72 | 0.626667 | 0 | 0 |
| 72 | 72 | 0.586667 | 0.083333 | 1 |
| 72 | 72 | 0.82 | 0.25 | 0 |
| 72 | 72 | 0.553333 | 0.333333 | 2 |
| 72 | 72 | 0.626667 | 0.5 | 0 |
| 72 | 72 | 0.906667 | 0.833333 | 0 |
| 72 | 72 | 0.873333 | 1 | 0 |
| 73 | 73 | 0.82 | 0.25 | 0 |
| 73 | 73 | 0.626667 | 0.5 | 0 |
| 74 | 74 | 0.413333 | 0.5 | 13 |
| 74 | 74 | 0.68 | 0.666667 | 8 |
| 74 | 74 | 0.726667 | 0.916667 | 80 |
| 74 | 74 | 0.76 | 1 | 25 |
| 75 | 75 | 0.633333 | 0.083333 | 6 |
| 75 | 75 | 0.773333 | 0.416667 | 2 |
| 75 | 75 | 0.8 | 0.583333 | 0 |
| 75 | 75 | 0.646667 | 0.75 | 0 |
| 75 | 75 | 0.346667 | 0.833333 | 1 |
| 75 | 75 | 0.9 | 1 | 6 |
| 76 | 76 | 0.713333 | 0.083333 | 8 |
| 76 | 76 | 0.753333 | 0.25 | 2 |
| 76 | 76 | 0.806667 | 0.416667 | 8 |
| 76 | 76 | 0.7 | 0.583333 | 4 |
| 76 | 76 | 0.686667 | 0.75 | 3 |
| 76 | 76 | 0.766667 | 1 | 11 |
| 77 | 77 | 0.726667 | 0.083333 | 12 |
| 77 | 77 | 0.746667 | 0.25 | 9 |
| 77 | 77 | 0.733333 | 0.416667 | 6 |
| 77 | 77 | 0.706667 | 0.583333 | 10 |
| 77 | 77 | 0.686667 | 0.75 | 18 |
| 77 | 77 | 0.853333 | 0.916667 | 0 |
| 77 | 77 | 0.846667 | 1 | 0 |
| 78 | 78 | 0.586667 | 0.583333 | 1 |
| 78 | 78 | 0.766667 | 0.666667 | 12 |
| 79 | 79 | 0.713333 | 0.083333 | 5 |
| 79 | 79 | 0.74 | 0.25 | 7 |
| 79 | 79 | 0.726667 | 0.416667 | 4 |
| 79 | 79 | 0.706667 | 0.583333 | 4 |
| 79 | 79 | 0.673333 | 0.75 | 0 |
| 80 | 80 | 0.533333 | 0 | 3 |
| 80 | 80 | 0.833333 | 0.25 | 2 |
| 80 | 80 | 0.693333 | 0.333333 | 0 |
| 80 | 80 | 0.64 | 0.5 | 2 |
| 81 | 81 | 0.573333 | 0.083333 | 25 |
| 81 | 81 | 0.76 | 0.25 | 10 |
| 81 | 81 | 0.706667 | 0.5 | 0 |
| 81 | 81 | 0.753333 | 0.666667 | 5 |
| 81 | 81 | 0.866667 | 0.833333 | 11 |
| 81 | 81 | 0.94 | 1 | 2 |
| 82 | 82 | 0.726667 | 0 | 11 |
| 82 | 82 | 0.66 | 0.083333 | 6 |
| 82 | 82 | 0.653333 | 0.25 | 8 |
| 82 | 82 | 0.56 | 0.416667 | 3 |
| 82 | 82 | 0.673333 | 0.583333 | 18 |
| 82 | 82 | 0.513333 | 0.666667 | 5 |
| 82 | 82 | 0.5 | 0.75 | 3 |
| 82 | 82 | 1 | 0.916667 | 6 |
| 83 | 83 | 0.806667 | 0.416667 | 2 |
| 83 | 83 | 0.5 | 1 | 0 |
| 84 | 84 | 0.573333 | 0 | 5 |
| 84 | 84 | 0.66 | 0.166667 | 11 |
| 84 | 84 | 0.746667 | 0.333333 | 9 |
| 84 | 84 | 0.826667 | 0.5 | 7 |
| 85 | 85 | 0.793333 | 0 | 18 |
| 85 | 85 | 0.566667 | 0.75 | 0 |
| 85 | 85 | 0.84 | 0.833333 | 0 |
| 85 | 85 | 0.88 | 0.916667 | 0 |
| 86 | 86 | 0.606667 | 0 | 0 |
| 86 | 86 | 0.673333 | 0.166667 | 0 |
| 86 | 86 | 0.7 | 0.333333 | 0 |
| 86 | 86 | 0.626667 | 0.5 | 0 |
| 86 | 86 | 0.713333 | 0.666667 | 0 |
| 86 | 86 | 0.733333 | 0.833333 | 0 |
| 86 | 86 | 0.813333 | 0.916667 | 2 |
| 86 | 86 | 0.826667 | 1 | 0 |
| 87 | 87 | 0.62 | 0 | 6 |
| 87 | 87 | 0.74 | 0.166667 | 27 |
| 87 | 87 | 0.546667 | 0.333333 | 6 |
| 87 | 87 | 0.813333 | 0.5 | 16 |
| 87 | 87 | 0.673333 | 0.666667 | 31 |
| 87 | 87 | 0.753333 | 0.833333 | 12 |
| 88 | 88 | 0.673333 | 0 | 9 |
| 88 | 88 | 0.613333 | 0.25 | 17 |
| 88 | 88 | 0.6 | 0.416667 | 18 |
| 88 | 88 | 0.766667 | 0.583333 | 93 |
| 88 | 88 | 0.706667 | 0.75 | 1 |
| 89 | 89 | 0.626667 | 0 | 0 |
| 89 | 89 | 0.586667 | 0.083333 | 0 |
| 89 | 89 | 0.626667 | 0.5 | 19 |
| 89 | 89 | 0.76 | 0.666667 | 19 |
| 89 | 89 | 0.74 | 0.833333 | 35 |
| 89 | 89 | 0.96 | 0.916667 | 33 |
| 90 | 90 | 0.666667 | 0.166667 | 0 |
| 90 | 90 | 0.953333 | 0.333333 | 1 |
| 90 | 90 | 0.893333 | 0.75 | 0 |
| 90 | 90 | 0.986667 | 1 | 0 |
| 91 | 91 | 0.426667 | 0 | 1 |
| 91 | 91 | 0.666667 | 0.166667 | 1 |
| 91 | 91 | 0.546667 | 0.333333 | 0 |
| 91 | 91 | 0.76 | 0.5 | 0 |
| 91 | 91 | 0.606667 | 0.666667 | 1 |
| 91 | 91 | 0.766667 | 0.833333 | 0 |
| 91 | 91 | 0.82 | 0.916667 | 0 |
| 92 | 92 | 0.633333 | 0.083333 | 1 |
| 92 | 92 | 0.686667 | 0.25 | 0 |
| 92 | 92 | 0.646667 | 0.416667 | 1 |
| 92 | 92 | 0.666667 | 0.583333 | 0 |
| 92 | 92 | 0.64 | 0.75 | 1 |
| 92 | 92 | 0.733333 | 0.916667 | 0 |
| 92 | 92 | 0.813333 | 1 | 3 |
| 93 | 93 | 0.653333 | 0.333333 | 0 |
| 93 | 93 | 0.673333 | 0.5 | 0 |
| 93 | 93 | 0.66 | 0.666667 | 3 |
| 93 | 93 | 0.646667 | 0.833333 | 1 |
| 93 | 93 | 0.34 | 1 | 0 |
| 94 | 94 | 0.673333 | 0.5 | 1 |
| 94 | 94 | 0.646667 | 0.833333 | 0 |
| 94 | 94 | 0.493333 | 0.916667 | 0 |
| 95 | 95 | 0.506667 | 0 | 1 |
| 95 | 95 | 0.626667 | 0.166667 | 2 |
| 95 | 95 | 0.686667 | 0.416667 | 0 |
| 95 | 95 | 0.633333 | 0.583333 | 0 |
| 95 | 95 | 0.786667 | 0.75 | 1 |
| 96 | 96 | 0.553333 | 0.166667 | 1 |
| 96 | 96 | 0.506667 | 0.833333 | 0 |
| 97 | 97 | 0.666667 | 0.083333 | 6 |
| 97 | 97 | 0.506667 | 0.833333 | 0 |
| 98 | 98 | 0.64 | 0.666667 | 0 |
| 98 | 98 | 0.68 | 1 | 4 |
| 99 | 99 | 0.653333 | 0 | 0 |
| 99 | 99 | 0.68 | 0.166667 | 0 |
| 99 | 99 | 0.666667 | 0.333333 | 1 |
| 99 | 99 | 0.693333 | 0.5 | 0 |
| 99 | 99 | 0.68 | 0.583333 | 0 |
| 99 | 99 | 0.673333 | 0.666667 | 4 |
| 99 | 99 | 0.606667 | 0.833333 | 2 |
| 99 | 99 | 0.82 | 0.916667 | 0 |
| 99 | 99 | 0.8 | 1 | 0 |
| 101 | 101 | 0.5 | 0.083333 | 4 |
| 100 | 100 | 0.62 | 0.25 | 2 |
| 101 | 101 | 0.586667 | 0.416667 | 4 |
| 101 | 101 | 0.486667 | 0.583333 | 1 |
| 101 | 101 | 0.613333 | 0.75 | 2 |
| 102 | 102 | 0.54 | 0 | 3 |
| 102 | 102 | 0.793333 | 0.166667 | 69 |
| 102 | 102 | 0.64 | 0.333333 | 2 |
| 102 | 102 | 0.68 | 0.5 | 3 |
| 102 | 102 | 0.666667 | 0.666667 | 6 |
| 102 | 102 | 0.673333 | 0.833333 | 1 |
| 103 | 103 | 0.633333 | 0.083333 | 2 |
| 103 | 103 | 0.686667 | 0.25 | 2 |
| 103 | 103 | 0.646667 | 0.416667 | 0 |
| 103 | 103 | 0.666667 | 0.583333 | 0 |
| 103 | 103 | 0.64 | 0.75 | 0 |
| 103 | 103 | 0.673333 | 1 | 3 |
| 104 | 104 | 0.953333 | 0 | 1 |
| 104 | 104 | 0.633333 | 0.833333 | 0 |
| 104 | 104 | 0.873333 | 0.916667 | 1 |
| 105 | 105 | 1 | 0.916667 | 8 |
| 105 | 105 | 0.98 | 1 | 71 |
| 106 | 106 | 0.853333 | 0.25 | 2 |
| 106 | 106 | 0.58 | 0.833333 | 0 |
| 106 | 106 | 0.92 | 0.916667 | 0 |
| 107 | 107 | 0.853333 | 0.25 | 0 |
| 107 | 107 | 0.293333 | 0.75 | 1 |
| 107 | 107 | 0.593333 | 0.916667 | 0 |
| 108 | 108 | 0.766667 | 0.666667 | 0 |
| 108 | 108 | 0.92 | 1 | 1 |
| 109 | 109 | 0.666667 | 0 | 14 |
| 109 | 109 | 0.76 | 0.166667 | 74 |
| 109 | 109 | 0.78 | 0.333333 | 46 |
| 109 | 109 | 0.753333 | 0.583333 | 14 |
| 109 | 109 | 0.78 | 0.75 | 28 |
| 109 | 109 | 0.446667 | 0.916667 | 21 |
| 109 | 109 | 0.686667 | 1 | 6 |
