# Supplementary material for: Improved Analysis of Long-Term Monitoring Data Demonstrates Marked Regional Declines of Bat Populations in the Eastern United States
Source: PLoS One. 2013 Jun 21;8(6):e65907. doi: 10.1371/journal.pone.0065907 (PMC3689752; doi:10.1371/journal.pone.0065907)
Supplement: Appendix S2 — Sample R code for GAMMs used in this study. (DOC) [file pone.0065907.s002.doc]

## Example R code

## the selected GAMM for M. lucifugus

MYLUgamm_1<<-gamm(LUCI_TOTAL~s(Winter_No,bs="cr",k=7) +

s(Day,bs="cr", k=10),family="poisson",

random=list(Location=~1, NAME=~1),

niter=1000

)

## display the AIC value

AIC (MYLUgamm_1$lme)

## Calculate projected trajectories and standard errors

## Create a dataframe on which to base predictions

pred.frame<-data.frame(Winter_No=c(0, 0.083333333, 0.166666667, 0.25, 0.333333333,

0.416666667, 0.5, 0.583333333, 0.666666667, 0.75, 0.833333333, 0.916666667, 1),

Day=rep(0.6842288,13))

## estimate predicted values

pc<-predict(MYLUgamm_1$gam,se.fit=TRUE,newdata=pred.frame,type="response")

pcE<-pc[["fit"]]

pcSE<-pc[["se.fit"]]

## concatenate results in a matrix alongside unscaled years

yrs<-c(1999:2011)

MaxFrac<-matrix(NA,13, 3)

for(i in 1:13){

MaxFrac[i,1]<-yrs[i]

MaxFrac[i,2]<-pcE[i]/max(pcE)

MaxFrac[i,3]<-2*(pcSE[i]/max(pcE))

}

## display the matrix

MaxFrac
