# Supplementary material for: Improved Analysis of Long-Term Monitoring Data Demonstrates Marked Regional Declines of Bat Populations in the Eastern United States
Source: PLoS One. 2013 Jun 21;8(6):e65907. doi: 10.1371/journal.pone.0065907 (PMC3689752; doi:10.1371/journal.pone.0065907)
Supplement: Appendix S3 — Tables of selected model terms for Day and Year for each bat species. (DOC) [file pone.0065907.s003.doc]

Appendix S3. Significance of model terms. Description of abbreviations: s() indicates a smoothed term, bs is the smoothing basis, cr indicates cubic regression splines, k is the maximum smoothing basis dimension, and edf is the estimated degrees of freedom.

*M. lucifugus*

Family: Poisson

Link function: log

Fixed effects:

LUCI_TOTAL ~ s(Winter_No, bs = "cr", k = 7) + s(Day, bs = "cr", k = 10)

Approximate significance of smooth terms:

|  | edf | F | p-value |
| --- | --- | --- | --- |
| s(Year) | 4.912 | 42.69 | < 2e-16 |
| s(Day) | 6.484 | 10.03 | 3.59e-11 |

*P. subflavus*

Family: Poisson

Link function: log

Fixed effects:

PIP_TOTAL ~ Winter_No

Approximate significance of linear terms:

|  | df | F | p-value |
| --- | --- | --- | --- |
| Year | 1 | 30.02 | 6.4e-08 |

*M. sodalis*

Family: quasipoisson

Link function: log

Formula:

SOD_TOTAL ~ Winter_No + s(Day, bs = "cr", k = 10)

Approximate significance of smooth terms:

|  | edf | F | p-value |
| --- | --- | --- | --- |
| Year | 1 | 18.68 | 2.16e-05 |
| s(Day) | 5.903 | 21.88 | < 2e-16 |

*M. septentrionalis*

Family: quasipoisson

Link function: log

Formula:

SEPT_TOTAL ~ Winter_No + s(Day, bs = "cr", k = 10)

Approximate significance of smooth terms:

|  | edf | F | p-value |
| --- | --- | --- | --- |
| Year | 1 | 3.895 | 0.049 |
| s(Day) | 4.342 | 2.926 | 0.0177 |
