# Supplementary material for: Improved Analysis of Long-Term Monitoring Data Demonstrates Marked Regional Declines of Bat Populations in the Eastern United States
Source: PLoS One. 2013 Jun 21;8(6):e65907. doi: 10.1371/journal.pone.0065907 (PMC3689752; doi:10.1371/journal.pone.0065907)
Supplement: Appendix S4 — Proportion of maximum expected value by year for each bat species. (DOC) [file pone.0065907.s004.doc]

Appendix S4. Population estimates and confidence intervals. Population estimates from the final model for each species for years 1999-2011. Values are the fraction of maximum observed value, so that the maximum population equals one.

| Year | *M. lucifugus* | *P. subflavus* | *M. sodalis* | *M. septentrionalis* |
| --- | --- | --- | --- | --- |
| 1999 | 0.95 (±0.35) | 1.00 (±0.60) | 1.00 (±0.35) | 1.00 (±0.26) |
| 2000 | 0.94 (±0.33) | 0.97 (±0.57) | 0.97 (±0.33) | 0.97 (±0.25) |
| 2001 | 0.93 (±0.33) | 0.93 (±0.55) | 0.94 (±0.31) | 0.94 (±0.24) |
| 2002 | 0.96 (±0.34) | 0.90 (±0.53) | 0.91 (±0.30) | 0.91 (±0.23) |
| 2003 | 0.99 (±0.35) | 0.87 (±0.51) | 0.89 (±0.28) | 0.88 (±0.23) |
| 2004 | 1.00 (±0.36) | 0.84 (±0.49) | 0.86 (±0.27) | 0.86 (±0.22) |
| 2005 | 0.96 (±0.34) | 0.81 (±0.47) | 0.84 (±0.26) | 0.83 (±0.21) |
| 2006 | 0.86 (±0.31) | 0.79 (±0.46) | 0.81 (±0.26) | 0.80 (±0.20) |
| 2007 | 0.73 (±0.26) | 0.76 (±0.44) | 0.79 (±0.25) | 0.78 (±0.20) |
| 2008 | 0.59 (±0.21) | 0.73 (±0.42) | 0.77 (±0.25) | 0.76 (±0.19) |
| 2009 | 0.46 (±0.16) | 0.71 (±0.41) | 0.74 (±0.25) | 0.73 (±0.19) |
| 2010 | 0.37 (±0.13) | 0.69 (±0.40) | 0.72 (±0.26) | 0.71 (±0.18) |
| 2011 | 0.29 (±0.11) | 0.66 (±0.38) | 0.70 (±0.26) | 0.69 (±0.18) |
